# How Non-invasive *in vivo* Cell Tracking Supports the Development and Translation of Cancer Immunotherapies

**DOI:** 10.3389/fphys.2020.00154

**Published:** 2020-04-03

**Authors:** Madeleine Iafrate, Gilbert O. Fruhwirth

**Affiliations:** Imaging Therapy and Cancer Group, Department of Imaging Chemistry and Biology, School of Biomedical Engineering & Imaging Sciences, King’s College London, London, United Kingdom

**Keywords:** adoptive cell therapy, cell tracking, drug development, molecular imaging, multi-modal whole-body imaging, positron emission tomography, reporter gene

## Abstract

Immunotherapy is a relatively new treatment regimen for cancer, and it is based on the modulation of the immune system to battle cancer. Immunotherapies can be classified as either molecular or cell-based immunotherapies, and both types have demonstrated promising results in a growing number of cancers. Indeed, several immunotherapies representing both classes are already approved for clinical use in oncology. While spectacular treatment successes have been reported, particularly for so-called immune checkpoint inhibitors and certain cell-based immunotherapies, they have also been accompanied by a variety of severe, sometimes life-threatening side effects. Furthermore, not all patients respond to immunotherapy. Hence, there is the need for more research to render these promising therapeutics more efficacious, more widely applicable, and safer to use. Whole-body *in vivo* imaging technologies that can interrogate cancers and/or immunotherapies are highly beneficial tools for immunotherapy development and translation to the clinic. In this review, we explain how *in vivo* imaging can aid the development of molecular and cell-based anti-cancer immunotherapies. We describe the principles of imaging host T-cells and adoptively transferred therapeutic T-cells as well as the value of traceable cancer cell models in immunotherapy development. Our emphasis is on *in vivo* cell tracking methodology, including important aspects and caveats specific to immunotherapies. We discuss a variety of associated experimental design aspects including parameters such as cell type, observation times/intervals, and detection sensitivity. The focus is on non-invasive 3D cell tracking on the whole-body level including aspects relevant for both preclinical experimentation and clinical translatability of the underlying methodologies.

## Introduction

Immunotherapy is a relatively new concept that is increasingly applied to a variety of conditions. Most of the currently approved or emerging immunotherapy approaches are in the oncology arena. In some cases, they were curative, which represents a major leap over most previous treatment concepts. Mechanistically, they modulate the immune system to better attack the cancer. There are two types of anti-cancer immunotherapy, molecular and cell-based immunotherapy. Both approaches are already in clinical use, whereby molecular immunotherapies currently are further developed with more applications and more approved therapeutics.

Molecular immunotherapies usually modulate the immune system by targeting immune checkpoints using antibodies or antibody-derived molecules. Examples include ICIs targeted at CTLA-4 (e.g. ipilimumab) or the PD-1/PD-L1 axis (e.g. nivolumab, atezolizumab, and pembrolizumab) (Hodi et al., 2010; Topalian et al., 2012; Larkin et al., 2015; Darvin et al., 2018). These immunotherapeutics were largely developed using similar regulatory approval frameworks to other receptor-targeting drugs. Although in several cases the whole-body distribution of these therapeutics would be accessible through imaging the molecular immunotherapy itself, this is not routinely performed. Only very recently did studies report the whole-body distribution of radiolabeled checkpoint inhibitors in man (e.g. atezolizumab, Bensch et al., 2018; Jauw et al., 2019) to assess whether imaging them might reveal prognostic information. Despite molecular immunotherapies changing the landscape of cancer treatment (Ledford et al., 2018), significant challenges remain. These include non-responding patients (Feng et al., 2013), severe immune-related adverse events (IrAE, i.e., ICI weakening the normal physiological barriers against autoimmunity resulting in various local and systemic autoimmune responses), and the development of resistance (Darvin et al., 2018).

Cell-based immunotherapies consist of live immune cells that are administered to patients. The anti-tumor properties are either intrinsic to these therapeutic cells or conferred to them through genetic engineering. The therapeutic immune cells are either taken from a different human donor (allogeneic) or are isolated from the patient (autologous) before undergoing manipulations that transform the cells into immunotherapeutic cells. A historic lack of clarity surrounding the regulatory aspect of live cell-based therapy resulted in debates on what constitutes manipulations requiring regulatory approval (Anon, 2014), but it is now accepted that any cells that have been cultured with any drugs are subject to regulatory approval. The new paradigm of cell-based immunotherapy has forced regulatory agencies to re-evaluate their approval processes to accommodate for living drugs and to avoid slowing progress; for example, the new ATMP framework accelerates the approval process if there is demonstrable clinical need (Marks and Gottlieb, 2018). The first ever clinically approved cell-based anti-cancer immunotherapies were the chimeric antigen receptor T-cell (CAR-T) therapies tisagenlecleucel and axicabtagene ciloleucel, both of which are autologous CD19-targeted CAR-T immunotherapies for the treatment of certain hematological malignancies (B-cell lymphomas; U.S. Food and Drug Administration, 2017). While spectacular treatment successes have been reported for CAR-T immunotherapies, alike molecular immunotherapeutics, not all patients responded and sometimes the effects were only temporary (Neelapu et al., 2017; Schuster et al., 2017; Maude et al., 2018), and these therapeutics have also been associated with severe side-effects and fatalities during trials (Linette et al., 2013; Saudemont et al., 2018). In addition, CAR-T immunotherapy has generally yielded disappointing results in solid tumors (Martinez and Moon, 2019). Nonetheless, the portfolio of immune cells envisaged for cell-based anti-cancer immunotherapy is increasing and now includes T-cell receptor-modified T-cells (TCR-T), γδ T-cells, NK and dendritic cells (DC). Importantly, there are several unknowns including the *in vivo* distribution, persistence and survival of cell-based immunotherapies as well as their efficacy at target and non-target sites, and there is a need to investigate these aspects during their development and translation into the clinics.

## The Need for Imaging in Immunotherapy Development

During the early stages of drug development, animal models are frequently employed to investigate the efficacies of drug candidates in defined disease settings. For instance, multiple animal tumor models have been used in the development of chemotherapeutics and targeted therapies (Cekanova and Rathore, 2014). Similar experimentation has also been necessary for the development of immunotherapies to establish targeting efficiencies, pharmacokinetics/pharmacodynamics, whether there is spatial heterogeneity to therapy delivery, and whether therapy presence is related to efficacy. Novel and accurate biomarkers are also essential to guide immunotherapy development to ensure optimal benefit for cancer patients. Notably, imaging biomarkers differ from conventional tissue/blood-based biomarkers in several important aspects (O’Connor et al., 2017). Foremost, imaging biomarkers are non-invasive, thus overcoming sampling limitations and associated tissue morbidities of conventional tissue/blood biomarkers, and they provide whole-body information albeit usually for only one target at the time. Furthermore, dynamic imaging can provide pharmacokinetic information. As with other biomarkers, imaging biomarkers should be standardized across multiple centers to unleash their full potential for diagnosis, patient stratification and treatment monitoring. Pathways for the development and standardization of dedicated imaging biomarkers have been structured and excellently described by a large team of cancer researchers (O’Connor et al., 2017), and we refer the reader to this publication for specific details.

**FIGURE 1:**
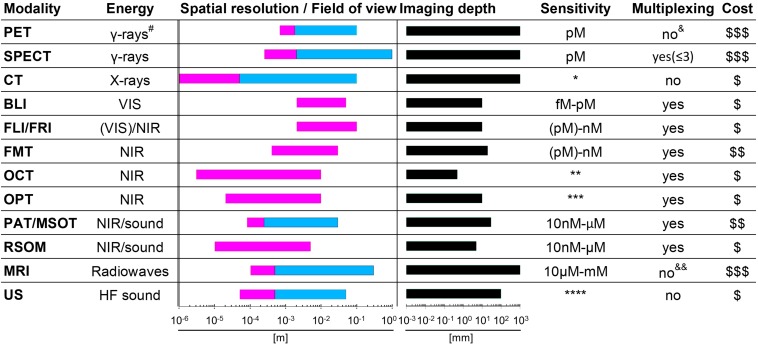
Properties of various whole-body imaging modalities. Imaging modalities are ordered according to the electromagnetic spectrum they exploit for imaging (top, high energy; bottom, low energy). Routinely achievable spatial resolution (left end) and fields of view (right end) are shown in red. Where bars are blue, they overlap red bars and indicate the same parameters but achievable with instruments used routinely in the clinic. Imaging depth is shown in black alongside next to sensitivity ranges. Instrument cost estimations are classified as ($) < 125,000 $, ($$) 125-300,000 $ and ($$$) > 300,000 $. ^#^Generated by positron annihilation (511keV). *Contrast agents sometimes used to obtain different anatomical/functional information. **In “emission mode” comparable to other fluorescence modalities (∼nM). ***Fluorophore detection can suffer from photobleaching by excitation light. ****Highly dependent on contrast agent. ^&^ Dual isotope PET is feasible but not routinely in use; it requires two tracers, one with a positron emitter (e.g. ^18^F and ^89^Zr) and the other with a positron-gamma emitter (e.g. ^124^I, ^76^Br, and ^86^Y), and is based on recent reconstruction algorithms to differentiate the two isotopes based on the prompt-gamma emission (Andreyev and Celler, 2011; Cal-Gonzalez et al., 2015; Lage et al., 2015). ^&⁣&^Multichannel MRI imaging has been shown to be feasible (Zabow et al., 2008). PET, positron emission tomography; SPECT, single photon emission computed tomography; CT, computed tomography; BLI, bioluminescence imaging; FLI, fluorescent lifetime imaging; FRI, fluorescent reflectance imaging; FMT, fluorescence molecular tomography; OCT, optical coherence tomography; OPT, optical projection tomography; PAT, photoacoustic tomography; MSOT, multispectral optoacoustic tomography; RSOM, raster-scan optoacoustic mesoscopy; MRI, magnetic resonance imaging; US, ultrasound.

Whole-body *in vivo* imaging technologies (Figure 1) that can interrogate cancers and therapeutics in preclinical models are very valuable tools in this context. They show great potential to provide answers to various challenges central to immunotherapy:

(1) Which immune cell classes are present in tumors and are they critical for response?
(2) What role do other components of the tumor microenvironment play?
(3) What are the consequences of heterogeneity within tumors and between lesions?
(4) What are biomarkers of true response and true progression?
(5) What is the relationship between target expression levels, affinity, and response?
(6) Can resistance be detected early or even be predicted?
(7) How can the distribution, fate, persistence and efficacy of cell-based immunotherapies be tracked *in vivo*?
(8) Can off-target effects and associated toxicities be detected early or be predicted?
(9) How can combination treatments be designed in a rational and effective manner?

Given that metastasis is responsible for >90% of cancer mortality, novel immunotherapy treatments need also to be evaluated for their efficacies against secondary lesions. Metastases can significantly differ from the primary tumor because of tumor evolution, and consequently can show a different therapy response compared to the primary lesion (Caswell and Swanton, 2017; Kim et al., 2019). While anti-metastatic endpoints have long been regarded impractical, it is noteworthy that an anti-metastatic prostate cancer drug, apalutamide, recently received FDA approval on the basis of metastasis-free survival as a new endpoint (measuring the length of time that tumors did not spread to other parts of the body or that death occurred after starting treatment, U.S. Food and Drug Administration, 2018). This raised the prospects for further such research, not least in the context of immunotherapy, and if immunotherapy were to be used as a treatment at earlier stages of cancer. Thus, preclinical models of metastasis employing *in vivo* traceable cancer cells have also a role to play in the development of immunotherapies (Gomez-Cuadrado et al., 2017).

## Imaging Approaches in Immunotherapy Development

### Brief Overview of Relevant Imaging Technologies

Medical imaging revolutionized the diagnosis and treatment of human disease by providing anatomical, physiological and molecular information (Mankoff, 2007). Imaging technologies differ in their capabilities and limitations. Figure 1 details the properties of those imaging technologies relevant to this review. Notably, several modalities are already in routine clinical use, for example US, magnetic resonance imaging (MRI), the radionuclide imaging modalities SPECT and PET, and X–ray computed tomography (CT). PAT and MSOT are two closely related relatively new modalities and have recently been translated into the clinical for special applications. PAT/MSOT delivers near infrared laser pulses into biological tissues with the latter absorbing and converting some of the laser pulse energy into heat, leading to transient thermoelastic expansion and thus wideband ultrasonic emission, which is used to compute an image (Ntziachristos et al., 2005; Wang and Yao, 2016). A purely optical imaging approach that is currently used in the clinical setting is OCT with applications in ophthalmology (Jung et al., 2011; Tao et al., 2013) and dermatology (Mogensen et al., 2009; Olsen et al., 2015). In general, the various imaging technologies can be categorized into modalities that subject the patient to a radiation dose (CT, PET, and SPECT) and modalities that are employing non-ionizing radiation (MRI, OCT, PAT/MSOT, and US). Depending on the research/clinical question, CT, MRI, PAT/MSOT, and US can be used with or without a contrast agent. In contrast, PET and SPECT strictly require contrast agents for image formation; these are often termed radiotracers, not least in reference to the very small concentrations required (“tracer levels;” picomolar concentration range) as both PET and SPECT are orders of magnitude more sensitive than the other clinically useable imaging technologies (Figure 1).

In preclinical settings, BLI can compete in sensitivity with radionuclide imaging modalities, but at much reduced experimental complexity and cost (instrument cost and running cost), which renders it a widely used tool. It relies on the presence of luciferase reporter proteins, which convert an administered chemical substrate into light that is then collected by highly sensitive cameras. As luciferase proteins are of non-mammalian origin, BLI is not translatable to the human setting. Another disadvantage is that BLI relies on light emitted within tissues which in turn is subject to absorption and scatter within the tissue matrix, thereby precluding reliable 3D quantification (Li et al., 2013; Dunlap, 2014; Jiang et al., 2016). Fluorescence-based whole-body imaging has also been developed (FLI/FRI), whereby fluorescence light is generated within a thick samples/small animal through excitation light; the approach has the same issues as BLI but is far less sensitive. To obtain true 3D data a tomographic design is required. Among the optical modalities listed in Figure 1, this is provided by optical projection tomography (OPT), which can be considered as the optical analog of CT. OPT operates on the micrometer to millimeter scales (Sharpe et al., 2002; Cheddad et al., 2012) thereby bridging the scale gap between classical whole-body imaging technologies and microscopy. It can either provide tomographic data on light absorption or fluorescence signals, and has been used in live zebrafish (Bassi et al., 2011; McGinty et al., 2011), fruit flies (Vinegoni et al., 2008; Arranz et al., 2014) and for whole organ imaging in mice (Alanentalo et al., 2008; Gleave et al., 2012; Gupta et al., 2013). An alternative approach offering larger fields of view in the centimeter range is diffuse optical tomography or FMT, which exploits photon tissue propagation theory to allow for 3D reconstruction at centimeter depth but its resolution is affected by weak signals and high tissues scattering (Figure 1; Graves et al., 2004; Ntziachristos, 2006; Venugopal et al., 2010; Zacharakis et al., 2011; Wang et al., 2015; Lian et al., 2017). In this review we lay emphasize on methodologies that are providing reliable quantifiable 3D information and have the potential to be clinically translatable.

As imaging modalities differ in their capabilities and limitations (Figure 1), combination technologies have become particularly important. For example, PET offers excellent sensitivity and provides absolute quantitative data (Lajtos et al., 2014) but can only detect signals at millimeter resolution. Hence, PET imaging was combined with other modalities providing higher anatomical resolution, such as CT (Basu et al., 2014) or MRI (Catana, 2017). This resulted in multi-modal whole-body imaging approaches adding anatomical context (from CT, MRI) to molecular imaging information (e.g. from PET or SPECT). Very recently, ultrafast US was combined with PET technology to form a new hybrid technology with the potential to provide molecular, anatomical and functional imaging data (Provost et al., 2018). Multi-modal imaging technologies are extremely useful to obtain maximal information from imaging, whereby the recent work of Bensch et al. provides a very good example for its power in the context of immunotherapy (Bensch et al., 2018).

### The Role of Anatomical Imaging

Anatomical imaging methods such as computed tomography (CT) or MRI provide excellent 3D resolution *in vivo* and enable quantification of tumor size and growth if the tumor differs sufficiently in contrast from surrounding tissues. Importantly, these techniques are non-specific and do not quantify tumor or immune cells specifically, but can account for the entirety of the tumor mass or reveal parameters such as texture (Lambin et al., 2012). This can cause issues for treatment monitoring if tumor size or radiomic features are not correlated to treatment response. If efficacy assessment is based on tumor shrinkage (cf. RECIST criteria in humans, Eisenhauer et al., 2009), then anatomical imaging is not appropriate for the assessment of immunotherapeutics, which initially can cause tumor sizes to increase or plateau before tumor regression occurs. This phenomenon is termed “pseudo-progression” and is evident in both molecular and cell-based immunotherapeutics (Nishino et al., 2017). It is caused by the very mechanisms of immunotherapy, which re-educates the immune system to detect and attack cancer cells, thereby resulting in immune cell infiltration/expansion, and tumors initially enlarging rather than regressing. Pseudo-progression has been recognized and is being accounted for in new criteria relevant for immunotherapy monitoring (Wolchok et al., 2009; Seymour et al., 2017).

### Molecular Imaging and Immunotherapy

**FIGURE 2:**
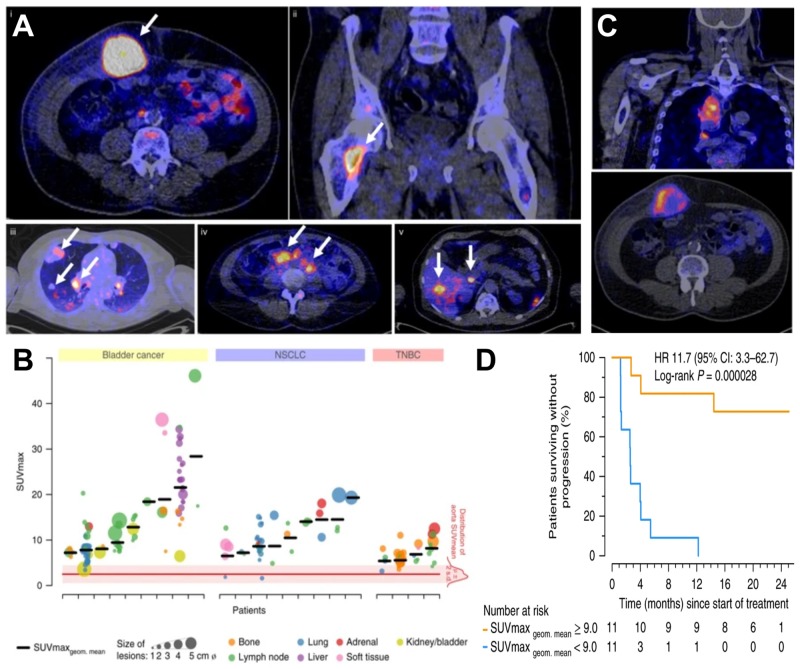
Molecular imaging can be used as a non-invasive tool to predict clinical response of immunotherapy. **(A)** Examples of PET/CT images of four patients illustrating ^89^Zr-atezolizumab tumor uptake in five different locations on day 7 post-contrast agent administration (white arrows indicate tumor lesions; PET scans were performed once per patient and time point). Images (i) and (ii) are from the same patient, whereas images (iii), (iv), and (v) are each from a separate patient. **(B)** Overview of ^89^Zr-atezolizumab uptake as SUV_max_ at day 7 post-contrast agent administration in 196 tumor lesions with a diameter >2 cm grouped per tumor type and ordered by increasing geometric mean SUVmax per patient, visualizing tumor size and site, and with the distribution of aorta for blood pool background uptake as reference. Horizontal bars indicate geometric mean SUVmax per patient. **(C)** PET/CT images of lesions of two patients with heterogeneous intralesional ^89^Zr-atezolizumab uptake on day 7 post contrast agent administration. *(Top)* Mediastinal lesion of a NSCLC patient (SUV_max_ 19.9) and *(Bottom)* abdominal wall metastases of a bladder cancer patient (SUV_max_ 36.4). **(D)** Progression-free survival according to the geometric mean standard uptake value (SUV_max_) per patient obtained by non-invasive PET imaging using ^89^Zr-labeled atezolizumab (orange, above-median geometric mean uptake; blue, below-median geometric mean uptake; *N* = 22 patients; two-sided log-rank test). For comparison, Hazard Ratios (HR) were only 2.6 and 1.3 for two different PD-L1 antibodies used in histology. For details see Bensch et al. (2018). (Reproduced with modifications from the indicated reference).

Molecular imaging differs from anatomical imaging in that it provides specific molecular information on the whole-body level. Molecular imaging can be exploited to visualize and quantify the presence of a target of interest at a given time on the whole-body level. This can be used to diagnose and guide patient stratification and treatment decisions. *Via* molecular imaging, the heterogeneity of target expression can be assessed, for example, between primary and secondary lesions or within individual tumors (Alizadeh et al., 2015; Kurland et al., 2017; Bensch et al., 2018). Importantly, molecular imaging can support treatment monitoring, for example, inform on target engagement, therapy efficacy, and in certain cases can be used to probe the activity of a therapeutic. Molecular imaging employs a broad variety of different contrast agent classes based on target-specific small molecules as well as a variety of biomolecules. The latter include full-length antibodies, bivalent F(ab’)_2_ fragments, minibodies, monovalent Fab fragments, diabodies, single-chain variable fragments (scFv), nanobodies, affibodies (listed in order of decreasing molecular weight). Strategies for developing and optimizing such targeted probes for non-invasive imaging using radioactive, optical, magnetic resonance, and ultrasound approaches have been recently summarized by Freise and Wu (Freise and Wu, 2015). The imaging of T-cell effector molecules such as the PD-L1/PD1 axis has been shown to be a successful approach to study T-cell *in vivo* distribution in preclinical models (Natarajan et al., 2015; Heskamp et al., 2019) and in humans (Jauw et al., 2019). However, a caveat of molecular imaging is its reliance on one chosen molecular target, because its expression might change during tumor progression, and with these changes also the imaging read-outs would change. Recently, the predictive power of molecular imaging for treatment outcome was demonstrated through visualization of the radiolabeled ICI atezolizumab by multimodal PET/CT imaging (combined molecular and anatomical imaging, Bensch et al., 2018; Figure 2). In this study, clinical responses were better correlated with pre-treatment [^89^Zr]Zr-desferrioxamine (DFO)-atezolizumab PET signals than with immunohistochemistry- or RNA-sequencing-based predictive biomarkers. [^89^Zr]Zr-DFO-pembrolizumab, which targets PD-1 on T-cells, is currently being tested in clinical trials involving non-small cell lung cancer or metastatic melanoma patients (NCT02760225, NCT03065764). Similarly, the T-cell expressed ICI target CTLA-4 has been imaged in preclinical mouse models of colon cancer to better understand target expression and therapy side effects (Higashikawa et al., 2014). Additionally, ^89^Zr-labeled ipilimumab targeting CTLA-4 in humans is in phase II trials (NCT03313323) to better comprehend the pharmacodynamics/pharamacokinetics of this antibody-based immunotherapeutic and its IrAEs. Other imaging targets related to T-cell effector functions include interferon-γ and granzyme B, which have both been studied in mice (Larimer et al., 2017, 2019; Gibson et al., 2018).

In preclinical models, the use of reporter genes to detect cancer cells *in vivo* (see Section “Non-invasive Whole-Body *in vivo* Cell Tracking”) can overcome specificity issues of anatomical imaging. Cancer cell tracking by means of reporter gene imaging is frequently performed using bioluminescence technology which is cost-effective and fast but suffers from the limitations of optical imaging, which preclude accurate quantification (Figure 1, see Section “Brief Overview of Relevant Imaging Technologies”). A recent article comparing BLI alone with combined BLI and radionuclide imaging demonstrated such aspects providing real-life examples in the context of cancer cell tracking (Vandergaast et al., 2019). It is noteworthy that other imaging technologies can fare well in some specialized cases. For example, metastasis tracking of melanin-producing murine melanoma cells was achieved in mice at reasonable sensitivity and resolution compared to the study aims by using PAT (Lavaud et al., 2017). Alternatively, radionuclide cancer cell tracking methodologies have been developed but they are more expensive and lower throughput techniques but provide 3D tomographic and fully quantitative information (e.g. Fruhwirth et al., 2014; Diocou et al., 2017; Volpe et al., 2018). The latter is currently being tackled by the development of multi-animal radionuclide imaging beds (e.g. four-mouse hotels, Greenwood et al., 2019).

## *In vivo* Imaging of T-Cell Populations

Specific cell surface markers on T-cells are attractive imaging targets as they enable the *in vivo* visualization of either all T-cells or distinct T-cell sub-populations. They can also be exploited for the quantification of therapeutic responses affecting T-cell presence (or absence) in cancerous tissues. For example, targeting T-cell receptors (TCR) is attractive because due to their high turnover on the plasma membrane, the bound radiotracers can gradually accumulate within the T-cells. In one preclinical study, TCRs were targeted using a ^89^Zr-conjugated anti-murine TCR F(ab’)2 fragment selective for the murine TCR beta domain. Using PET/CT imaging, this radiotracer was shown to track the location of adoptively transferred engineered T-cells *in vivo*; notably, imaging data and *ex vivo* quantification of transgenic T-cell numbers in tumors correlated well (Yusufi et al., 2017). Additionally, using a ^64^Cu-labeled anti-chicken OVA-TCR antibody, it was demonstrated that associated TCR internalization neither impaired antigen recognition *via* the TCR, nor did it diminish T-cell viability or function in mice (Griessinger et al., 2015).

Alternatively, targeting CD3, a T-cell surface glycoprotein and pan-T-cell marker, has been suggested. Therefore, a radiometal-chelated antibody against CD3 ([^89^Zr]Zr-DFO-CD3) was designed to quantify T-cell infiltration during anti-CTLA-4 treatment in colon cancer xenograft models. As the host species were mice, a murine anti-CD3 antibody was required in this case, and large amounts of infiltrated T-cells were found in the tumor prior to regression (Larimer et al., 2016). The LAG-3 was similarly exploited to image T-cells in xenografts established in transgene mice expressing human LAG-3 as host strains (Kelly et al., 2018). The radiolabelling strategy also utilized the siderophore-based chelator DFO, and this ^89^Zr-based radiotracer is now in clinical development. Moreover, ^89^Zr-LAG-3 PET is currently investigated in patients suffering from head and neck cancer or non-small cell lung cancer (NCT03780725). Notably, both anti-CD4 and anti-CD8 cys-diabodies have been radiometal-labeled (^89^Zr, ^64^Cu) to track the corresponding T-cell sub-populations in preclinical models. Using these radiotracers, researchers imaged treatment responses of immunotherapies, for example response to checkpoint inhibitors such as anti-PD-1 (Seo et al., 2018) or anti-PD-L1 (alone or in combination with adoptive cell therapy) (Tavare et al., 2016; Freise et al., 2017; Zettlitz et al., 2017). The advantage of incorporating antibody fragments rather than full-length antibodies into the design of *in vivo* imaging agents is that the end product will reach its target more quickly, and it will be excreted faster (Bates et al., 2019). Such engineered antibody fragments targeting CD8 have already progressed into clinical trials (NCT03107663, NCT03802123, and NCT03610061). To increase specificity and reduce liver toxicity and Fc γ receptor binding, bispecific antibodies targeting both T-cells (e.g. *via* 4-1BB) and either tumor antigens (e.g. CD19) or tumor stroma (e.g. FAP) have been developed. The bispecific antibodies have also been conjugated to radioisotopes to track their *in vivo* distribution in rodents by SPECT or PET imaging (Claus et al., 2019).

The mentioned approaches are applicable to the development of various molecular immunotherapies and cell-based immunotherapies. A general limitation is that the obtained imaging signals cannot be used to back-calculate precise T-cell numbers because the precise expression levels of T-cell surface marker molecules are unknown at the point of imaging. All above described methods probe T-cell presence but not their activities. As for cell-based immunotherapies, there is an additional limitation, namely the lack of discrimination between adoptively transferred and resident cells. To overcome this the adoptively transferred cells would need to be labeled to distinguish them from the resident ones (cf. Section “Non-invasive Whole-Body *in vivo* Cell Tracking”).

## Imaging the Activation of T-Cells

Upon antigen-recognition and co-stimulation, naïve T-cells become activated in secondary lymphoid organs, which results in the expression of various cell surface markers of T-cell activation. The latter can be imaged using specific antibodies or antibody-fragments. For example, OX40 (CD134/TNFRSF4) is such a cell surface-expressed marker of T-cell activation and it has been used to image the spatiotemporal dynamics of T-cell activation following *in situ* vaccination with CpG oligodeoxynucleotide in a dual tumor-bearing mouse model (Alam et al., 2018). Moreover, it was shown that OX40 imaging using ^64^Cu-DOTA-AbOX40 as a contrast agent for PET predicted tumor responses with greater accuracy than both blood-based measurements for early response (i.e., Luminex analyses including interferon-γ, tumor necrosis factor α, MCP1, MIP1B etc.) and anatomical measurements in this mouse model. Another example is the trimeric IL -2 receptor (CD25/IL-2Ra), which was exploited to visualize activated T-cells in immune-compromised mice by PET imaging using the contrast agent *N*-(4-[^18^F]fluorobenzoyl)- IL -2 (Di Gialleonardo et al., 2012). IL-12 has also been implicated as a specific target for T-cell activation. Consequently, ^99m^Tc-labeled IL-12 has been used to detect T-cell activation *in vivo* in mice, albeit in colitis and not yet in tumor models (Annovazzi et al., 2006). Moreover, bioluminescence and radionuclide imaging tools to assess TCR-specific activation of T-cells have been developed (Ponomarev et al., 2001; Kleinovink et al., 2018), however, these approaches are still in preclinical development and are based on genetic engineering of T-cells and thus constitute specialized variants of cell tracking as described in Section “Non-invasive Whole-Body *in vivo* Cell Tracking”.

Alternatively, it is possible to exploit the observation that T-cells undergo metabolic changes upon activation in tissues (van der Windt and Pearce, 2012; Buck et al., 2016) resulting in the influx of substrates not normally present in non-activated T-cells. While targeting metabolic pathways with imaging agents can distinguish activated from non-activated T-cells, this approach can suffer from competing signals generated by different cells in close vicinity. A very promising PET tracer in this context is 2′-deoxy-2′-[^18^F]fluoro-9-β-D-arabinofuranosylguanine ([^18^F]F-AraG), which accumulates in activated T-cells predominantly *via* two salvage kinase pathways (Ronald et al., 2017). Notably, the unlabeled compound has previously been used as a T-cell depleting drug in refractory T-cell acute lymphoblastic leukemia. [^18^F]F-AraG PET imaging in a murine acute graft-vs.-host-disease (GvHD) model enabled visualization of secondary lymphoid organs harboring activated donor T-cells prior to clinical symptoms of GvHD. Notably, the biodistribution of [^18^F]F-AraG was favorable and it may be useful for imaging activated T-cells in the context of immunooncology, which is currently investigated in several clinical trials (NCT03311672, NCT03142204, and NCT03007719).

## Non-Invasive Whole-Body *in vivo* Cell Tracking

The exploitation of molecular imaging has also enabled spatiotemporal whole-body *in vivo* tracking of administered cells (Kircher et al., 2011). One form of *in vivo* cell tracking has long been used to localize occult infections in patients (de Vries et al., 2010; Roca et al., 2010). Technological and methodological advances over the last decade led to a resurgence of cell tracking, this time in conjunction with the emergence of live cell therapeutics. For their development, several important questions remain largely elusive and require attention;

(i) the whole-body distribution of therapeutic cells;
(ii) their potential for re-location during treatment and the kinetics of this process;
(iii) whether on-target off-site toxicities occur;
(iv) how long the administered cells survive; and
(v) which biomarkers are best suited to predict and monitor cell therapy efficacy.

Traditional approaches in preclinical cell therapy development relied on dose escalation with toxicity evaluation, tumorigenicity tests, and qPCR-based persistence determination. Whole-body imaging-based *in vivo* cell tracking can inform on questions (i)-(iv) of these aspects in a truly non-invasive manner. However, many clinical trials are still performed largely without knowledge of the *in vivo* distribution and fate of the administered therapeutic cells, making it impossible to adequately monitor and assess their safety, thereby raising ethical questions when considering complications in clinical trials that could have been averted or mitigated if whole-body imaging had been used (Linette et al., 2013; Saudemont et al., 2018). With cell-based anti-cancer immunotherapies currently centered on adoptively transferred T-cells, either subjected to genetic engineering or *ex vivo* expansion only, there was a need to develop corresponding imaging tools to quantify T-cells *in vivo* on the whole-body level.

### Methods of *in vivo* Cell Tracking

*In vivo* cell tracking rests on the principles and mechanisms of molecular imaging to achieve contrast between cells of interest and the other cells of the organism. In some cases, there are intrinsic features of the cells of interest that can be exploited for generating contrast, for example, when cells produce targetable molecules that show low or no expression in other tissues. Under these circumstances, conventional molecular imaging offers cell tracking possibilities both preclinically and clinically. Examples demonstrating this are; tracking thyroid cancer metastases using the NIS (Kogai and Brent, 2012; Portulano et al., 2014), exploiting the PSMA to image prostate cancer and its spread (Perera et al., 2016; Oliveira et al., 2017), carcinoembryonic antigen (CEA) for colorectal cancer imaging (Tiernan et al., 2013), or melanin imaging in melanomas (Tsao et al., 2012). However, in most *in vivo* cell tracking scenarios, including all reported cases of cell-based immunotherapy, contrast agents or contrast-generating features must be introduced to the cells of interest. Fundamentally, cell labels can be introduced to cells *via* two different methodologies, direct or indirect cell labeling.

#### Direct Cell Labeling for Cell-Based Immunotherapies

**FIGURE 3:**
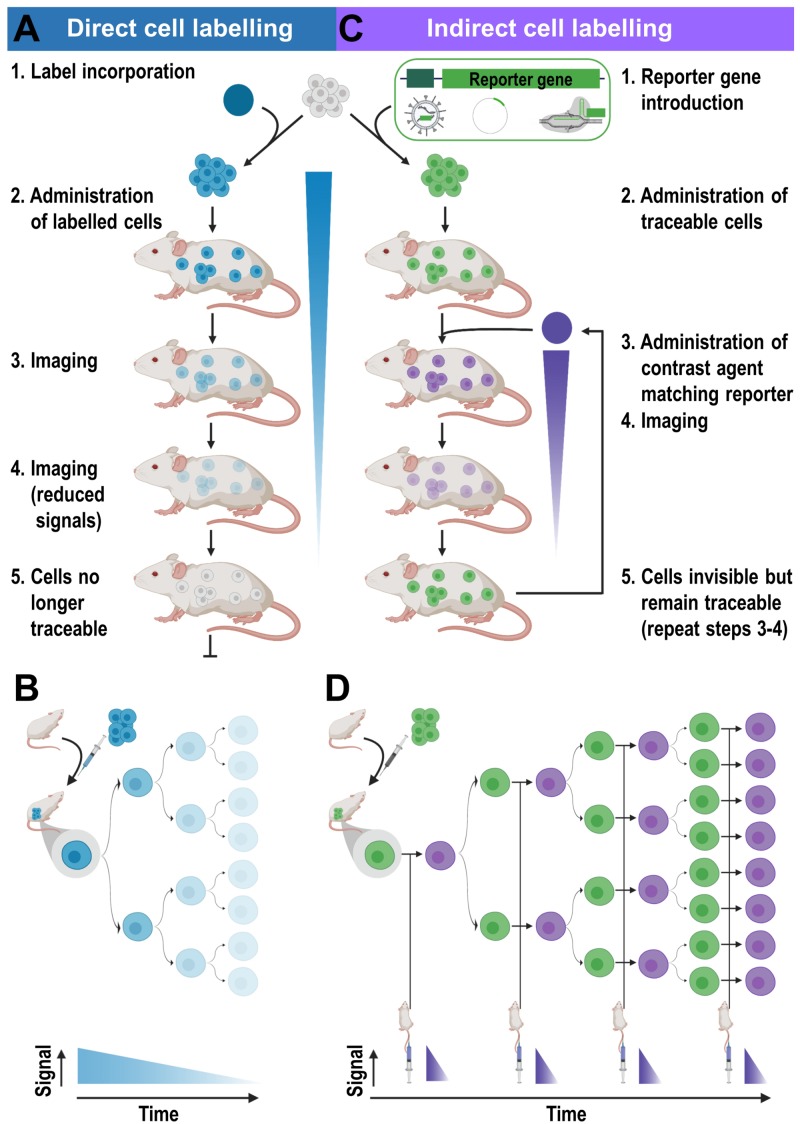
Cell labeling approaches and their consequences for *in vivo* detectability of cells. **(A)** Cells are directly labeled by incorporation of a contrast agent (blue) matching the desired imaging technology. Cells can either take up the contrast agent on their own (e.g. through phagocytosis, *via* internalizing receptors etc.) or are labeled through assisted contrast agent uptake (e.g. cell permeant contrast agents, transfection etc.). The labeled cells (blue) are administered to animals and remain traceable until the contrast agent concentration per cell becomes too dilute to be detectable. Several processes including label efflux, label dilution through cell division, and in the case of radioisotopes also radioactive decay contribute and limit the maximum observation time *in vivo*. **(B)** Scheme depicting the effects of label dilution on cell detectability. **(C)** Indirect cell labeling requires the incorporation of a reporter gene (green) under the control of a suitable promoter (dark green). Reporter genes are frequently introduced using viruses but can also be incorporated *via* episomal plasmids or gene editing. Engineered cells (green) are administered to animals and can be visualized *in vivo via* administration of corresponding contrast agents (purple) followed by imaging, which can be repeated to enable long-term tracking. **(D)** Filial generations of reporter gene expressing cells remain traceable, hence indirectly labeled cells are *in vivo* traceable indefinitely.

Direct cell labeling is performed upon cells *ex vivo*, and the subsequently labeled cells are re-administered into subjects, where they can be tracked using the relevant imaging technology (Figure 3A). Cells can either take up the contrast agents on their own (e.g. through phagocytosis, *via* internalizing receptors etc.) or are labeled through assisted contrast agent uptake (e.g. using cell permeant contrast agents, transfection etc.). There is a large variety of ready-to-use contrast agents available including chelated radiometals (for PET or SPECT), ^19^F-fluorinated nanoparticles and iron oxide nanoparticles (for various MRI types), as well as organic fluorophores and fluorescent nanoparticles (for optical imaging); for more details the reader is referred to a recent review by Kircher et al. (2011). One strength of MRI imaging is its excellent whole-body resolution. Consequently, various nanoparticles have been used to label and track adoptively transferred cells in preclinical models by MRI (Qiu et al., 2018). When applied to cell-based immunotherapy in humans, ^19^F-fluorinated nanoparticles have been proven effective cell-tracking contrast agents for MRI (Srinivas et al., 2013), as ^19^F is naturally almost absent in tissues. Unfortunately, the detection sensitivity of ^19^F is very low and requires specialized equipment. Attempts to improve detection sensitivities included the use of molecules and nanoparticles incorporating many ^19^F atoms (Srinivas et al., 2012). Longitudinal tracking of activated T-cells *in vivo* was reported for a period of nearly three weeks in mice (Srinivas et al., 2009), but others found only limited utility for *in vivo* tracking of similarly labeled CD4^+^/CD8^+^ T-cells (in a murine diabetes model, Saini et al., 2019). Despite multiple optical contrast agents available for cell labeling, whole-body *in vivo* cell tracking using optical methodologies is very limited. This is caused by the intrinsic shortcomings of optical imaging including high tissue absorption and scatter precluding accurate *in vivo* localization and quantification. This includes 3D fluorescence molecular tomography (FMT), which also suffers from poor resolution, limited depth penetration and low sensitivity compared to other modalities (Figure 1). In the following, we focus on direct cell labeling with radioisotopes, because radionuclide imaging is currently the most sensitive tool for *in vivo* tracking of directly labeled cells in mammals. When co-registered with CT or MRI for additional anatomical detail, SPECT/PET-MRI/CT images are most promising to aid clinical translation of cell-based immunotherapies. Radioisotope can be used for cell labeling by either (i) exposing cells to chelating agents such as radiometal-complexed hydroxyquinolines (oxines) resulting in cellular uptake *via* diffusion or transport-mediated processes, or by (ii) linking radioisotopes onto cell surfaces, either electrostatically (e.g. using cell insertion peptides) or covalently.

In inflammatory conditions and infectious disease, the radiometal chelators [^111^In]In-oxine and [^99m^Tc]Tc-HMPAO have been routinely used clinically for tracking *ex vivo* labeled cells, e.g. white blood cells (Roca et al., 2010; de Vries et al., 2010). This decades-old methodology has more recently been applied to clinical studies of CD4^+^ T-cells in Hodgkin’s lymphoma (Grimfors et al., 1989), to assess penetrance of tumor-infiltrating lymphocytes in melanoma (Fisher et al., 1989; Griffith et al., 1989) or autologous CD8^+^ T-cells in early stage non-small cell lung cancer patients who receive anti-PD-L1 immunotherapy in a neo-adjuvant setting (NCT03853187). Both ^111^In and ^99m^Tc are compatible with SPECT imaging or scintigraphy, an imaging technology which has previously been shown to be insufficiently sensitive in clinical studies (James and Gambhir, 2012). Although technological advances in SPECT instrumentation are improving the situation somewhat, PET imaging remains the method of choice, since it offers absolute quantification and higher sensitivity on clinical instrumentation. The PET isotope equivalent of ^111^In (τ = 2.8 days) is ^89^Zr (τ = 3.3 days), which has a similar half-life but different decay properties (^89^Zr: 23% positron emission, higher energy γ–rays than ^111^In but lacking Auger electron emission). Like ^111^In, cell labeling with ^89^Zr became possible with oxine chelators (Charoenphun et al., 2015; Sato et al., 2015), and it was shown to be better retained inside cells than [^111^In]In-oxine (Charoenphun et al., 2015). This is a major advantage because the images correspond to the locations of the radioisotope; if labels leak out of cells rapidly they are more likely to give unreliable results. [^89^Zr]Zr-oxine has been widely applied preclinically for immune cell labeling of cytotoxic T lymphocytes (CTL), γδ T-cells, DC and CAR-T (Sato et al., 2015; Weist et al., 2018; Man et al., 2019). With GMP-compatible protocols now available, [^89^Zr]Zr-oxine is on a trajectory toward clinical translation and ultimately it will replace [^111^In]In-oxine, which has become increasingly scarce in the EU due to economic reasons (Dhawan and Peters, 2014). A limitation of PET is its restricted spatial resolution (Figure 1) which is fundamentally limited by the radioisotope-dependent average positron range in matter (Phelps et al., 1975; Cal-Gonzalez et al., 2013). A way to mitigate its low resolution is to combine it with anatomical imaging methods that feature higher resolution (PET/MRI and PET/CT). For cell tracking applications also nanoparticle-based, multimodal PET/MRI probes have been envisaged, for example iron oxide nanoparticles that are cross-linked to radioisotopes (Garcia et al., 2015).

An alternative direct cell labeling methodology is to link contrast agents to the cell surface of cells. For example, [^89^Zr]Zr-DFO-NCS was used to label human mesenchymal stem cells and while retained on the cell surface for about a week, cell viability appeared to be unaffected (Bansal et al., 2015). This approach is constrained by the availability of cell surface reactive groups that can be exploited, in this case primary amines, and it has the potential to interfere with cell surface proteins and impair cell function. This could restrict its use, particularly if tracer-level concentrations are superseded to achieve high *ex vivo* cell labeling to expand the cell tracking time (cf. Figures 3A,B). However, no systematic comparative studies between cell uptake and cell surface linking of radiometals in lymphocytes have so far been reported.

#### Indirect Cell Labeling Applied to Immunotherapy Development

**FIGURE 4:**
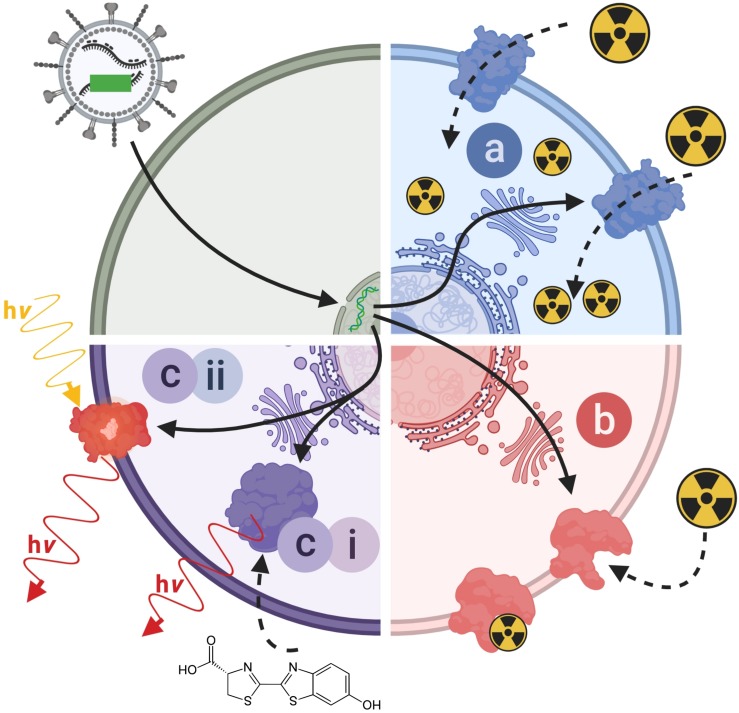
Molecular imaging mechanisms relevant to reporter genes for indirect cell labeling. Cartoon showing the three main molecular imaging mechanism that are exploited for indirect cell labeling. **(a)** Transport (blue): these reporters are expressed at the plasma membrane of cells and each expressed reporter can transport several contrast agent molecules into the cell, which constitutes a signal amplification mechanism. The radionuclide transporters NIS and NET belong to this class of reporters. **(b)** Protein binding (red): these reporters are also normally expressed at the plasma membrane of cells and contrast agents bind directly to them; minor levels of signal amplification are theoretically possible if several contrast agents could bind to the reporter, or if several contrast agents could be fused to a reporter binding molecule; however, signal amplification is inferior compared to transporters. Examples for this reporter class are PSMA and SSTR2. **(c)** Contrast forming reporters (purple) can be sub-divided into two categories; enzymes that can generate contrast, and proteins that act as labels with intrinsic contrast. **(c_i_)** Enzymatic contrast formation: such reporters either entrap a molecular probe or generate a contrast agent from a precursor that needs to be either supplied externally or is available within the cell. Thymidine kinases such as HSV1-*tk* are examples for enzymes that entrap a radiotracer through its phosphorylation, and thereby generate contrast. Firefly luciferases are examples of reporters that convert an externally supplied substrate [shown: luciferin light (hν)]. Tyrosinase is an example of a reporter which converts cell-intrinsic precursors to the contrast agent melanin. **(c_ii_)** Intrinsic contrast: these reporters produce a signal on their own, normally upon stimulation. Classical examples are all fluorescent proteins, which generate specific light emissions upon excitation with light matching their excitation spectra. For details and literature references to relevant reporter genes see Tables 1, 2. NIS, sodium/iodide symporter; NET, norepinephrine transporter; PSMA, prostate specific membrane antigen; SSTR2, somatostatin receptor 2.

Indirect cell labeling is based on genetic engineering of cells to ectopically express a reporter, which serves as an imaging target (Figure 3C). This imaging target is then imaged *in vivo* after administration of suitable contrast agents, for example short half-life radiotracers, in a process that can be repeated to detect the traceable reporter-expressing cells over time (Figures 3C,D). Introduction of genetically encoded reporters is most frequently performed by viral transduction to ensure genomic integration and long-term expression. In some cases, episomal plasmids have been used (e.g. delivered by transfection or electroporation; Lufino et al., 2008; Ronald et al., 2013). Lately, gene editing approaches have been exploited for reporter insertion as they can be advantageous to viral transduction because they offer precise control over the genomic site of reporter insertion (Bressan et al., 2017). With feasibility having been demonstrated, this approach is likely to receive greater attention in the cell therapy field in future. Contrast formation relies on one of several mechanisms (Figure 4): either

**TABLE 1:** Reporter gene classes according to their molecular imaging mechanisms (cf. Figure 4) including selected examples.

**Mechanism [cf. Figure 4]**	**Reporter**	**Properties**	**Matching imaging modality**	**References**
Transporter [a]	Mammalian transporters	Sodium iodide symporter (NIS, SLC5A5); Norepinephrin transporter (NET, SLC6A2); Dopamine transporter (DAT, SLC6A3).	Various radiotracers for PET and SPECT for all reporters listed.	(Dai et al., 1996; Moroz et al., 2007; Jauregui-Osoro et al., 2010; Khoshnevisan et al., 2016, 2017; UCL Business PLC, 2017; Jiang et al., 2018)
	Ion transporter from magnetotatic bacteria	MS-1 magA.	MRI (Endogenous or exogenous iron).	(Nakamura et al., 1995; Zurkiya et al., 2008; Cho et al., 2014)
	Polypeptides	Sodium-Taurocholate Co-transporting Polypeptide (NTCP).	Fluorescence and MRI.	(Wu et al., 2019)
Cell surface protein binding [b]	G-protein-coupled receptors	Somatostatin receptor type 2 (SSTR2); Dopamine receptor (D_2_R).	PET and SPECT radiotracers available; PET radiotracers available.	(Satyamurthy et al., 1990; MacLaren et al., 1999; Rogers et al., 1999, 2000; Zinn et al., 2000a; Chaudhuri et al., 2001; Liang et al., 2001; Hwang et al., 2007)
	Recycling receptor	Transferrin receptor.	MRI (SPIO).	(Weissleder et al., 2000)
	Cell-surface antigen-based reporter	Human carcino-embryonic antigen-based reporters are recombinant proteins based on CEA minigene (N-A3) fused to extracellular and transmembrane domains of human FcγRIIb receptor, CD5 or TfR carboxyterminal domain.	PET and SPECT radiotracers available.	(Hammarstrom, 1999; Hong et al., 2008; Kenanova et al., 2009; Barat et al., 2011; Girgis et al., 2011)
	Mammalian cell surface protein	PSMA and mutants; radiotracers bind to the protein using it as a cell surface protein and not exploiting its enzymatic properties.	PET and SPECT radiotracers available.	(Castanares et al., 2014; Minn et al., 2019)
Enzymes [ci]	Bacterial enzymes	*E. coli* dihydrofolate reductase (eDHFR); *E. coli* β-galactosidase.	PET; Various including OPTICAL (chemiluminescence), MRI, PET and SPECT.	(Fowler and Zabin, 1977; Louie et al., 2000; Li et al., 2007; Liu and Mason, 2010; Green et al., 2017; Sellmyer et al., 2017, 2019; Guo et al., 2019; Krueger et al., 2019)
	Mammalian and non-viral kinases	Pyruvate kinase M2, thymidine kinases (viral such as HSV1-*tk* and mammalian variants), deoxycytodine kinases.	Various PET tracers for the individual kinases.	(Tjuvajev et al., 1995; Ponomarev et al., 2007; Jang et al., 2010, 2012; Likar et al., 2010; Park et al., 2015; Lee et al., 2017; Haywood et al., 2019; Seo et al., 2019).
	Other mammalian enzymes	Tyrosinase	PAT/MSOT, MRI, PET.	(Weissleder et al., 1997; Ponomarev et al., 2004; Krumholz et al., 2011)
	Luciferases	Various luciferases including Firefly, Green Click Beetle; Gaussia, Renilla; and NanoLuc.	OPTICAL (bioluminescence): Firefly, Green Click Beetle: D-luciferin; Gaussia, Renilla: coeloenterazine; NanoLuc: imidazopyrazinone.	(Lorenz et al., 1991; Loening et al., 2006; Tannous, 2009; Inoue et al., 2011; Hall et al., 2012; Schaub et al., 2015; Germain-Genevois et al., 2016; La Barbera et al., 2017; Mezzanotte et al., 2014, 2017; Hunt et al., 2016; Aswendt et al., 2019; Weihs and Dacres, 2019; Zhang et al., 2019)
Fluorescent Proteins [cii]	Proteins with intrinsic fluorophores	Red fluorescent: E2-Crimson/mTagRFP/mPlum/mNeptune; Infrared fluorescent: iRFP 670/iRFP 720.	OPTICAL (fluorescence upon appropriate excitation): (emission λ_max_): 543/584/649/650; (emission λ_max_): 670/720.	(Merzlyak et al., 2007; Kremers et al., 2009; Lin et al., 2009; Filonov et al., 2011; Liu et al., 2013; Shcherbakova and Verkhusha, 2013; Deliolanis et al., 2014; Isomura et al., 2017; Zhou et al., 2018; Fukuda et al., 2019)
Frequency-selective contrast/other	Artificial protein	Contrast based on transfer of radiofrequency labeling from the reporter’s amide protons to water protons.	MRI (CEST).	(Gilad et al., 2007; Farrar et al., 2015)
Formation of gas vesicles/other	Mammalian acoustic reporter gene (mARG)	Gas vesicles are produced which generate US contrast.	US (3.2 MPa insonation).	(Farhadi et al., 2019)

**TABLE 2:** Promising host-compatible reporter genes and their corresponding imaging tracers.

**Reporter**			**Reporter *in vivo* detection**		
**Class**	**Name**	**Properties**	**Imaging modality and contrast agent**	**Contrast agent properties**	**References**
Transporter	Sodium iodide symporter (NIS)	Symports Na^+^ alongside various anions. Endogenous expression in thyroid, stomach, lacrimal, salivary and lactating mammary glands, small intestine, choroid plexus and testicles.	PET: ^124^I^–^, [^18^F]BF_4_^–^, [^18^F]SO_3_F^–^, [^18^F]PF_6_^–^. SPECT: ^99m^TcO_4_^–^, ^123^I^–^.	Tracers do not cross BBB.	(Dai et al., 1996; Jauregui-Osoro et al., 2010; Khoshnevisan et al., 2016, 2017; Jiang et al., 2018)
	Norepinephrine transporter (NET)	NaCl-dependent monoamine transporter. Endogenously expressed in organs with sympathetic innervation (heart, brain),	PET: [^124^I]MIBG**; [^11^C]hydroxyephedrine. SPECT: [^123^I]MIBG**.	Tracers do not cross BBB.	(Moroz et al., 2007)
	Dopamine transporter (DAT)	NaCl-dependent.	PET: [^11^C]CFT, [^11^C]PE2I, [^18^F]FP-CIT. SPECT: ^123^I-β-CIT**, ^123^I-FP-CIT**, ^123^I-Ioflupane**, ^99m^TRODAT.	Few data in public domain. Tracers cross BBB.	(UCL Business PLC, 2017)
Enzyme	Pyruvate kinase M2	Expression during development, also in cancers.	PET: [^18^F]DASA-23.	Background in organs of excretion route. Suggested for cell tracking within brain. Tracer crosses BBB.	(Haywood et al., 2019)
	Thymidine kinase (hmtk2/hΔTK2)	Human kinase causing cellular tracer trapping.	PET: [^124^I]FIAU**, [^18^F]FEAU, [^18^F]FMAU (for hTK2-N93D/L109F).	Tracers do not cross the BBB; Endogenous signals in gall bladder, intestine and organs involved in clearance.	(Ponomarev et al., 2007)
	Deoxycytidine kinase (hdCK)	Human kinase causing cellular tracer trapping.	PET: [^124^I]FIAU**, [^18^F]FEAU.	Tracers do not cross the BBB; Endogenous signals in gall bladder, intestine and organs involved in clearance.	(Likar et al., 2010; Lee et al., 2017)
Cell surface receptor	Somatostatin receptor type 2 (SSTr2)	G-protein-coupled receptor. Endogenous expression in brain, adrenal glands, kidneys, spleen, stomach and many tumors (i.e., SCLC, pituitary, endocrine, pancreatic, paraganglioma, medullary thyroid carcinoma, pheochromocytoma);	PET: ^68^Ga-DOTATOC, ^68^Ga-DOTATATE. SPECT: ^111^In-DOTA-BASS. (best tracers selected here).	Tracers may cause cell signaling, change proliferation and might inhibit impair cell function. Non-metal octreotide radiotracers can cross blood brain barrier (BBB).	(Rogers et al., 1999, 2000; Zinn et al., 2000a, b; Chaudhuri et al., 2001)
	Dopamine receptor (D_2_R)	G-protein-coupled receptor. High endogenous expression in pituitary gland and striatum.	PET: [^18^F]FESP, [^11^C]Raclopride, [^11^C]N-methylspiperone.	Slow clearance of [^18^F]FESP; Tracers cross BBB.	(Satyamurthy et al., 1990; MacLaren et al., 1999; Liang et al., 2001; Hwang et al., 2007)
	Transferrin receptor (TfR)	Fast recycling receptor.	MRI: Transferrin-conjugated SPIO.	Transferrin-conjugated SPIOs are internalized by cells.	(Weissleder et al., 2000)
Cell surface protein	Glutamate carboxy-peptidase 2 (PSMA) and variant tPSMA^N9del^	tPSMA^N9del^ has higher plasma membrane concentration. High expression in prostate.	PET: [^18^F]DCFPyL, [^18^F]DCFBC. SPECT: [^125^I]DCFPyL**.anti-PSMA antibodies and ligands can be flexibly labeled*, e.g. J951-IR800.	Background signal in kidneys. Tracers do not cross BBB.	(Castanares et al., 2014; Minn et al., 2019)
Cell surface antigen	Human carcino-embryonic antigen (hCEA)	Overexpressed in pancreatic, gastric, colorectal and medullary thyroid cancers.	PET: ^124^I-anti-CEA scFv-Fc H310A**, [^18^F]FB-T84.66 diabody SPECT: ^99m^Tc-anti-CEA Fab’ (approved), ^111^In-ZCE-025, ^111^In-anti-CEA F023C5i.	Tracers do not cross BBB.	(Griffin et al., 1991; Hammarstrom, 1999; Hong et al., 2008; Kenanova et al., 2009)
Artificial cell surface molecule	DOTA antibody reporter 1 (DAbR1)	ScFv of anti-DOTA antibody 2D12.5/G54C fused to human CD4 TM domain.	PET:^86^Y-AABD.	Tracer is a DOTA complex that binds irreversibly to a cysteine residue in the 2D12.5/G54C antibody. Tracer does not cross BBB.	(Wei et al., 2008)
	Estrogen receptor α ligand binding domain	No reported physiological function.	PET: [^18^F]FES.	Tracer is clinically used estrogen receptor imaging agent.	(Qin et al., 2013)
	Anti-PEG Fab fragment*	Some tracers cross BBB; PEG is non-toxic and approved by FDA.	PET: ^124^I-PEG-SHPP*,**. MRI: SPIO-PEG. Fluorescence: e.g. NIR797-PEG.	Iodine tracers bear risk of deiodination. Some tracers cross BBB.	(Chuang et al., 2010)
Carrier protein	Ferritin		MRI: iron.	Iron is not equally distributed across the brain and therefore may cause local susceptibility shifts that are above the MRI detection limit.	(Cohen et al., 2005; Genove et al., 2005)

Promise was evaluated based on (i) host-compatibility of the reporter and (ii) availability of contrast agents. *Any other modality can be used provided a suitable contrast forming moiety will be attached to PEG and the CEA antibodies, respectively. **Radioiodinated tracers can become de-iodinated *in vivo* resulting in free iodide that is subsequently taken up into NIS expressing organs.

(a) label uptake into cells by transporters,
(b) label binding to cell surface-expressed reporters, or
(c) expression of contrast-forming proteins, which either (i) produce a label through enzymatic action (e.g. luciferases, tyrosinase), or (ii) act as labels themselves (e.g. fluorescent proteins).

All these mechanisms can be useful for preclinical cell tracking and a variety of corresponding reporter genes are listed in Table 1. For clinical cell tracking, the emphasis must lies on the mechanisms (a) and (b), because the contrast-forming proteins are either not of human origin or produce toxic products if expressed outside their original context (e.g. tyrosinase; Urabe et al., 1994) and thus not clinically translatable. Alongside improvements of imaging technologies, also the corresponding reporter genes have been developed and optimized. A fundamental drawback of indirect cell labeling is that it requires genetic engineering. However, this is neither a concern for preclinical experimentation nor for cell therapies already reliant on it (e.g. CAR-T) (Saudemont et al., 2018). Several factors require careful consideration when planning reporter gene-afforded *in vivo* cell tracking experiments, particularly in the context of immunotherapies (see Section “Experimental Design Considerations for *in vivo* Cell Tracking”).

### Multiplex Cell Tracking

It would be highly beneficial to track both primary tumors and metastases alongside the therapeutic in preclinical models. Combining preclinical whole-body cancer cell tracking with imaging of molecular or cell-based immunotherapeutics could enable image-based quantification of the extent a labeled/traceable immunotherapy reaches *in vivo* traceable cancers, and whether the immunotherapy is delivered to all primary/secondary lesions. Dual-modality approaches would be required for this, ideally both tomographic in nature to enable the 3D quantification of metastasis burden alongside the immunotherapy. While almost every well-performed preclinical immunotherapy imaging study cross-correlates tumor targeting of the traceable therapeutic with either anatomical or molecular imaging in the primary tumor, metastases have rarely been accounted for. One example of such a study evaluating also the metastatic sites involved was performed by Edmonds *et al.* (Edmonds et al., 2016) in a preclinical breast cancer model. The authors employed dual-radioisotope imaging to co-track cancer metastases and a liposomally encapsulated immunomodulatory drug with the aim to optimize the time between liposome administration and the subsequent adoptive transfer of γδ T-cell immunotherapy involving both primary and secondary lesions. In a subsequent preclinical study, the same authors co-tracked ^89^Zr-oxine labeled γδ T-cells (direct labeling approach) to NIS reporter expressing breast cancer cells (indirect labeling approach using ^99m^TcO_4_^–^ as a NIS radiotracer) employing sequential multi-modal PET-SPECT-CT imaging (Man et al., 2019).

## Experimental Design Considerations for *in vivo* Cell Tracking

To ensure immunotherapy development benefits from cell tracking, it is imperative that the cell labeling approaches for therapeutic and/or cancer cells are chosen with the experimental goals in mind. Considerations must include a variety of different aspects such as cell tracking time, cell tracking interval, experimental setting (preclinical or clinical), use of immunocompetent or immunocompromised host organisms, imaging technology, and contrast agent properties and availability. To detect cells, the employed cell label must match the envisaged imaging technology to be used. The choice of the imaging technology dictates the achievable spatial resolution and imaging depth (Figure 1), impacts on minimal temporal resolution through image acquisition speeds, and contributes majorly to detection sensitivity and cost. Availability of the imaging technology and the necessary label further impact on the feasibility of collaborative across different institutions, which is of particular importance for clinical translation of a methodology and the chances of its subsequent adoption in clinical practice.

### Imaging Technology and Its Impact on Cell Detection Sensitivity

#### Considerations for the Selection of the Imaging Technology

Exquisite detection sensitivity is required for *in vivo* cell tracking applications. In practice, this means sensitivities should be within or below the picomolar concentration range (Figure 1), which can be achieved best with bioluminescence and radionuclide imaging modalities. Unlike radionuclide imaging technologies, BLI neither provides absolute quantitative data nor true 3D information and is applicable only preclinically. However, despite its shortcomings, BLI has so far been the most frequently used preclinical approach to measure the impact of immunotherapeutics on *in vivo* traceable bioluminescent tumors; most likely due to BLI being relatively cheap and fast. In special cases, BLI can currently provide unique information relevant to immunotherapy development on the preclinical level. For example, dual-luciferase reporter methodology enabled the quantification of *in vivo* T-cell activation in specifically engineered transgene mice (Mezzanotte et al., 2011; Kleinovink et al., 2018). While it is fundamentally possible to perform such preclinical experiments with more quantitative 3D radionuclide tomography, it has not been reported so far; most likely due to more complex logistics and higher costs associated with this approach (e.g. two different radiotracers with similar pharmacokinetics/pharmacodynamics would be needed for each individual imaging session).

In many cases, 3D tomographic whole-body imaging data is required in rodents or larger mammals, i.e., non-translucent organisms. This generally limits the use of optical imaging technologies due to their inherent limitations relating to tissue light absorption and scatter. Hence, radionuclide imaging modalities are preferred for such purposes, but they require dedicated reporter genes, which are scarce compared to the plethora of different fluorescent proteins or luciferases that have been developed (Thorn, 2017; Shcherbakova et al., 2018; Mezzanotte et al., 2017). If clinical translation is the main goal for cell tracking applications, then radionuclide imaging is the most suitable approach to address the questions raised in Section “Non-invasive Whole-Body *in vivo* Cell Tracking.”

#### Detection Sensitivity and the Duration of Cell Tracking

Detection sensitivity of labeled cells depends on the cellular label concentration and the matched imaging technology. The different labeling methodologies affect the cellular label concentration in different ways (see Section “Non-invasive Whole-Body *in vivo* Cell Tracking”). Label dilution, label efflux and in the case of radioactive labels dosimetry, can be severe limitations of direct cell labeling methodologies. The impact of label dilution has been discussed above (Section “Direct Cell Labeling for Cell-Based Immunotherapies”). Copper isotopes are the main example wherein label efflux causes issues. ^64^Cu (τ = 12 h) had been suggested as a shorter half-life PET isotope (Adonai et al., 2002; Li et al., 2009; Bhargava et al., 2009) potentially competing with the SPECT isotope ^99m^Tc (τ = 6.0 h). However, its unfavorably high cellular efflux (>50% per 4-5 h) paired with efficient liver uptake resulted in low signal-to-background ratios *in vivo* (Adonai et al., 2002; Bhargava et al., 2009; Li et al., 2009; Griessinger et al., 2014). High label efflux also limited the use of the long half-life PET radiometal ^52^Mn for cell tracking (Gawne et al., 2018). For considerations regarding dosimetry see Section “Impact of Cell Labeling Methodology on Cell Function.”

For indirectly labeled cells, the molecular imaging mechanism of the used reporter gene and the cellular expression level of the reporter gene are crucial, while the label dilution aspect plays no significant role (Figure 3D). Reporter genes which enzymatically entrap radiotracers that are taken up into cells offer high cell detection sensitivities. Examples are thymidine kinases, which phosphorylate and thereby entrap radiotracers in the cells, e.g. HSV1-*tk* is detected through its corresponding PET radiotracer [^18^F]FHBG. Transporters (e.g. NET or NIS) provide signal amplification as each reporter protein can transport several radiotracer molecules into the cell. It is noteworthy that ectopic expression of reporters can affect the fate of their substrates. For example, NIS is normally expressed in thyroid follicular cells and its regular substrate, iodide, is metabolized into thyroid hormones after cell import. Upon ectopic expression in non-thyroidal cells, e.g. cancer cells or immune cells, this downstream mechanism affecting the equilibrium of the imported iodide is non-existent resulting in iodide not being accumulated to the same extent compared to thyroid tissues. To apply radioiodide for cell tracking in humans, it would be necessary to counteract high thyroid uptake and radioiodide metabolization there as this could lead to thyroid damage. The latter is possible by prior administration of non-radioactive iodide, but this also impacts on detection sensitivity of the traceable cells of interest. Non-iodide NIS radiotracers, which are not metabolized and wash out of thyroid cells would thus be preferable. As NIS is not very selective regarding its anion substrates (Paroder-Belenitsky et al., 2011) anionic radiotracers that are roughly similar in size and shape were developed for NIS imaging; they include ^99m^TcO_4_^–^, [^18^F]BF_4_^–^, [^18^F]SO_3_^–^ or [^18^F]PF_6_^–^ (Jauregui-Osoro et al., 2010; Khoshnevisan et al., 2017; Jiang et al., 2018). They are not entrapped in cells, neither in thyroidal tissues nor in cells ectopically expressing NIS. Therefore, it would be advantageous to use non-iodide NIS radiotracers for clinical cell tracking. Another advantage of the new NIS PET radiotracers is that they are based on ^18^F, which has superior decay properties compared to ^124^I. ^18^F decays with a half-life of 109.8 min to ^18^O with 96.9% positrons (E_mean_ = 0.250 MeV and 0.6 mm average positron range), while ^124^I decays with a half-life of 4.18 days to ^124^Te with only 22.7% positrons (11.7% β_2_^+^ at E_mean_ = 975 MeV and 4.4 mm mean range, and 10.7% β_1_^+^ at E_mean_ = 0.687 MeV and mean 2.8 mm range, plus a minor 0.3% β_3_^+^ at 0.367 MeV and 1.1 mm mean range) accompanied by several γ–rays and a high proportion of electron capture (Conti and Eriksson, 2016). Consequently, ^18^F produces more positrons per decay resulting in better detectability. It is noteworthy that ^18^F positrons also have a lower mean energy than those of ^124^I resulting in lower mean positron ranges (until annihilation and emission of detectable γ–rays) and therefore enabling better PET resolution. While free positron range considerations are currently irrelevant for clinical PET imaging (instrument resolution with 3-4 mm larger than most average free positron ranges of relevant PET isotopes), they are of concern for preclinical PET imaging (instruments can provide resolution even below 1 mm with the right isotope) (Deleye et al., 2013; Nagy et al., 2013). Notably, first-in-man clinical studies using [^18^F]BF_4_^–^ to image NIS have already been completed (O’Doherty et al., 2017), thereby lowering the hurdles for NIS-afforded PET reporter gene imaging as a means of cell tracking in humans. Such considerations regarding the selection of radioisotopes as part of an individual reporter:contrast agent pair are transferable also to other reporters for which contrast agents with different radioisotopes are available (see Table 2). Selection of the best suited reporter:contrast agent pair is paramount.

**FIGURE 5:**
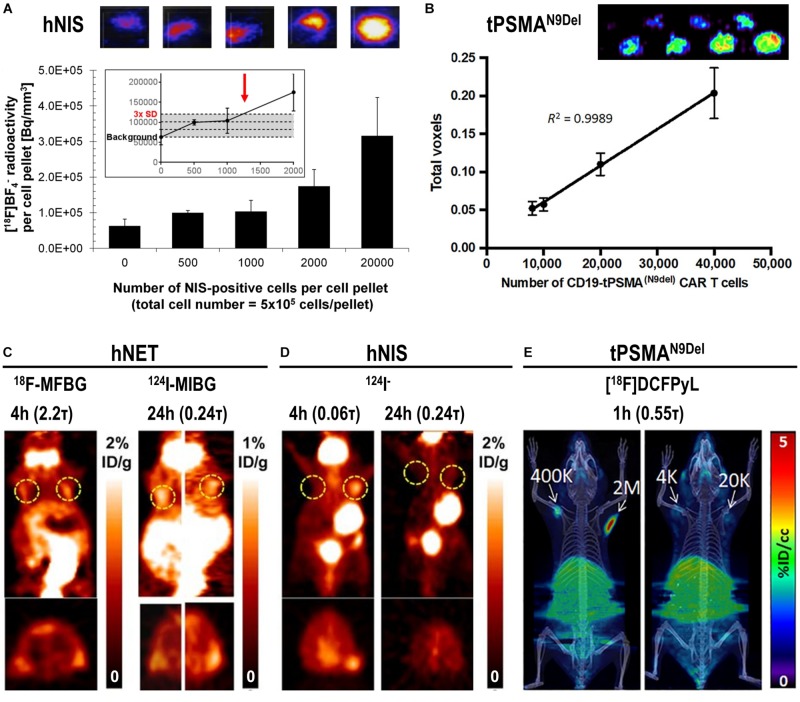
*In vitro* and *in vivo* detection sensitivity of reporter gene expressing cells. **(A)** *In vitro* determination of the detection limit of NIS-positive cells within a cell pellet of NIS-negative cells for the NIS radio tracer [^18^F]BF_4_^–^ using nanoPET/CT equipment from Mediso. For experimental details see Diocou et al. (2017) (*top*) Typical results of nanoPET/CT imaging of cell pellets and *(bottom)* quantitative analysis of imaging experiments. The limit of detection was determined to be ∼1,250 NIS-positive cells (inset, red arrow). **(B)** Standard curve demonstrating a linear relationship between the PET signal and the number of CD19-tPSMA^N9del^ CAR-T. *(top) In vitro* phantom from which the standard curve was derived. The *in vitro* phantom used varying numbers of CD19-tPSMA^(N9*del)*^ CAR T cells incubated with [^18^F]DCFPyL, a high affinity, positron-emitting ligand targeting PSMA; cell numbers were in the top row 10^3^, 2⋅10^3^, 4⋅10^3^, and 6⋅10^3^, and in the bottom row 8⋅10^3^, 10^4^, 2⋅10^4^, and 4⋅10^4^. Images were acquired using a SuperArgus small-animal PET/CT instrument from Sedecal. The data in the graph show results from the bottom row of images. The detection limit was determined to be around 2,000 cells. For experimental details see Minn et al. (2019). **(C,D)** PET *in vivo* imaging of human primary T-cells transduced with hNET **(C)** or hNIS **(D)** reporter genes. Different numbers of T-cells were injected subcutaneously, followed by systemic administration of indicated corresponding radiopharmaceuticals. PET imaging at indicated time points after radiotracer administration was performed using a Focus 120 microPET scanner from Siemens. Number of T-cells injected is (left dashed ring) 3⋅10^5^ and (right dashed ring) 10^6^. No potentially interfering signals were thresholded and data are expressed as percentage injected dose per gram (%ID/g). For experimental details see Moroz et al. (2015). **(E)** NSG mice injected with the indicated number of CD19-tPSMA^N9del^ CAR-T in 50 μL (50% Matrigel) in the shoulders (white arrows). Mice were *in vivo* imaged on the Sedecal’s SuperArgus small-animal PET/CT at 1 h after administration of the corresponding radiotracer [^18^F]DCFPyL. PET data are expressed in percentage of injected dose per cubic centimeter of tissue imaged (%ID/cc). To improve the display contrast of the *in vivo* images, relatively high renal radiotracer uptake was masked using a thresholding method. For experimental details see Minn et al. (2019). (Figure combined from the publications referenced in the legend above; permissions from corresponding publishers obtained).

The detection sensitivities of NIS-expressing extra-thyroidal cells have been reported preclinically to be as good as hundreds/thousands for cancer cells expressing NIS (Fruhwirth et al., 2014; Diocou et al., 2017) and CAR-T expressing PSMA *in vitro* (Minn et al., 2019), or tens of thousands for effector T-cells using various different reporter genes *in vivo* (Moroz et al., 2015) (Figure 5). Comparative studies aiming at the evaluation of how different reporter genes impact on T-cell detectability have been performed in the past (Moroz et al., 2015). Importantly, since this study new reporter gene:contrast agent pairs have become available, for example PSMA paired with its high-affinity PET ligand [^18^F]DCFPyL (Minn et al., 2019) or NIS paired with its PET radiotracer [^18^F]BF_4_^–^ (see above). Consequently, new comparative studies are needed to conclude on relative reporter:contrast agent performance in relevant immune cells; ideally performed such that also reporter expression levels and their intracellular availabilities for interaction with their contrast agents are precisely controlled. As reporter expression levels are cell type-dependent, it is highly recommended to determine detection sensitivities of indirectly labeled cells in each case and also on the available instrumentation before designing *in vivo* cell tracking experiments.

### Proliferation of Traceable Cells and *in vivo* Tracking Time

Paramount for choosing the cell labeling approach is how rapid the traceable cells divide and how long-term an observer wishes to track them *in vivo*. Tracking cancer cells in preclinical tumor models normally entails following them over multiple cell divisions, spreading over weeks if not months. To evaluate cell-based anti-cancer immunotherapies, cell engraftment, expansion and survival are of interest, whereby observation times, usually several days up to several weeks, are long-term compared to division/expansion events of therapeutic cells.

For direct cell labeling applications label efflux and label dilution are limiting (Figures 3B,D). If radioisotopes are used for direct cell labeling then their half-lives additionally limit achievable tracking times with 4-5 half-lives being realistic with existing small-animal PET instrumentation; e.g. about two weeks for ^89^Zr (Khoshnevisan et al., 2017) depending on the amount of radiolabel loading and instrument sensitivity. As the continued presence of the radioisotope in direct cell labeling results in a radiation dose to the cell and consequently radiation damage accumulation, a compromise must be reached between the maximum label concentration, which should not impair cell function (see Section “Impact of Cell Labeling Methodology on Cell Function”), and the maximum achievable tracking time.

In contrast, indirect cell labeling does not suffer from label dilution as the genetically encoded reporter is passed on to filial generations, thereby rendering the observation time theoretically indefinite. Indirect cell labeling relies on repeat administration of contrast agents, if radioactive then short half-live radioisotopes. This adds complexity as for example radiotracers need to be freshly prepared for every imaging session (Figure 3D), but it is certainly advantageous that the overall received doses are smaller than in direct cell labeling when compared over the same tracking periods. Consequently, indirect cell labeling is the preferred method of choice for long-term cell tracking, including cancer cell tracking in spontaneous metastasis models or the long-term evaluation of cell-based immunotherapies.

### *In vivo* Cell Tracking Interval

During tracking of directly labeled cells, the imaging interval is not linked to the imaging technology other than that animal welfare considerations must be considered (e.g. minimum interval of repeat-anesthesia). However, for indirect cell labeling approaches with radionuclide reporter gene and corresponding radiotracers, the choice of radioisotope affects the minimum imaging interval because the radiotracer from an earlier imaging session must have had time to sufficiently decay before a new batch can be administered to enable a subsequent imaging session (Figure 3D). For example, there are various radiotracers for the radionuclide reporter NIS, which have differing radioisotope half-lives; these include ^99m^TcO_4_^–^ (τ = 6.01 h) and ^123^I^–^ (τ = 13.2 h) for SPECT or ^124^I^–^ (τ = 101 h) and [^18^F]BF_4_^–^ (τ = 1.83 h) for PET. Again, ∼4-5 half-lives are needed for sufficient radiotracer decay and this defines the minimum time interval acceptable between imaging sessions. It was previously shown that repeat-imaging of NIS-expressing cells is possible after four half-lives using ^99m^TcO_4_^–^, i.e., after 24 h (Diocou et al., 2017); however, this would not be possible for over two weeks when using ^124^I^–^, while [^18^F]BF_4_^–^ would allow ∼8 h intervals.

As for radionuclide imaging-afforded cell tracking it is noteworthy that radiotracer concentrations are very low, generally below target saturation, hence presence of prior administered radiotracers is normally negligible for later imaging sessions due to very low picomolar radiotracer concentrations, even if they would stay intact and not be excreted. A special case in this context is ^99m^Tc, which decays to long-lived ^99^Tc and consequently remains in the same chemical form after its decay. Thus, it could accumulate over repeat imaging sessions unless excreted. For example, in preclinical NIS imaging experiments in mice the radiotracer ^99m^TcO_4_^–^ is administered at 15 – 30 MBq per animal. This equates to about 0.5 – 1.0 pmol of the total pertechnetate species (sum of ^99m^TcO_4_^–^ and ^99^TcO_4_^–^) taking into account a typical ^99m^TcO_4_^–^ generator elution regimen and ^99^TcO_4_^–^ carrier presence (Lamson et al., 1975). Even in an unrealistic worst-case scenario excluding its renal excretion, each repeat NIS imaging session would add this amount to the animal. However, the overall pertechnetate concentration would still be far below NIS saturation as its Michaelis-Menten constant for pertechnetate is likely very similar to those reported for ReO_4_^–^ or ClO_4_^–^ (Paroder-Belenitsky et al., 2011) and hence in the low micromolar range. Others repeatedly imaged animals with NIS-expressing cancer cells by ^99m^TcO_4_^–^ -SPECT and found no impact of earlier imaging sessions on subsequent ones (Diocou et al., 2017). For radiotracers that decompose chemically following radioisotope decay this consideration is irrelevant. Such an example is the NIS PET radiotracer [^18^F]BF_4_^–^, in which ^18^F decays to ^18^O resulting in a chemically instable product ultimately generating borate, which is no longer a substrate for NIS (Khoshnevisan et al., 2016). While presented using the example of NIS here, such considerations can also be relevant for other reporter gene:radiotracer pairs.

### Cell Viability and Its Impact on Detected Cell Tracking Signals

Signals from directly labeled cells do not report on whether the cells are alive. Moreover, recorded signals might not even stem from the initially labeled cell population (e.g. due to label efflux or cell death and subsequent deposition or uptake into different cells). In contrast, indirect cell labeling is fundamentally linked to cell viability as the reporter is encoded in the DNA of the traceable cells. However, signal loss in reporter expressing cells is also a possibility, for example, when the reporter gene expression cassettes become epigenetically silenced. Notably, so-called ‘safe harbor locations’ have been discovered in mammalian genomes (Pellenz et al., 2019), and reporter genes can be inserted into such locations using gene editing methodologies. The latter has recently be demonstrated in different stem cell types even with large reporter genes such as NIS (Wolfs et al., 2017; Ashmore-Harris et al., 2019).

**FIGURE 6:**
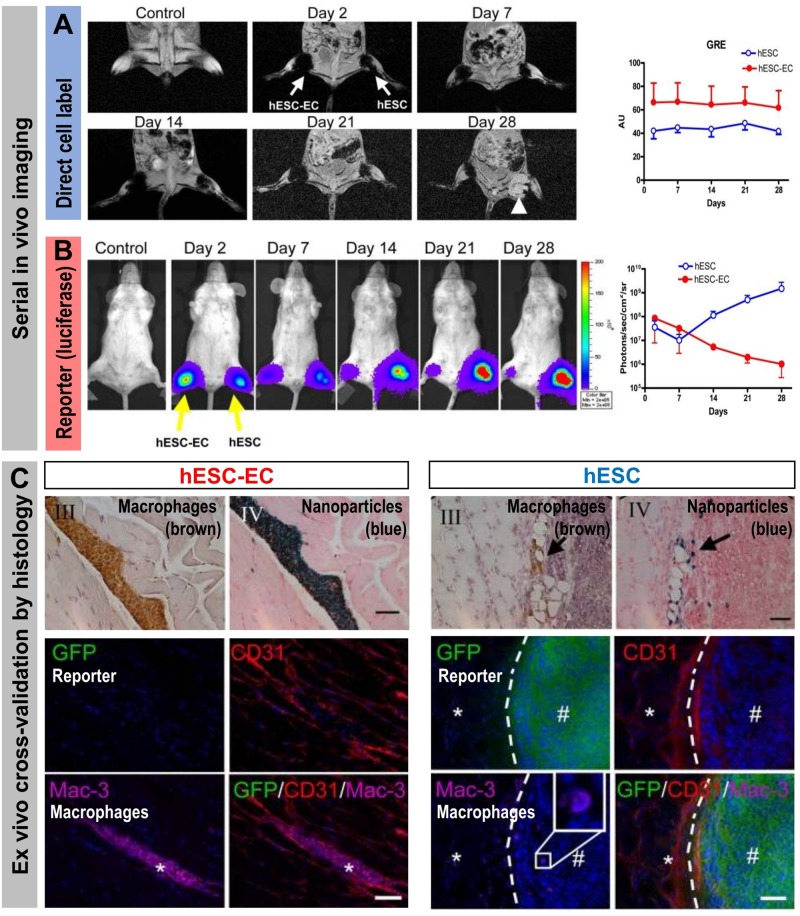
How the type of cell labeling impacts on conclusions drawn from signals obtained from serial imaging. Cell viability can be assessed better from indirectly labeled cells than from directly labeled cells as shown by a cross-validation study using both direct and indirect cell labeling within the same cells. Reporter gene (luciferase and GFP)-expressing human embryonic stem cells (hES) or human embryonic stem cells differentiated to endothelial cells (hESC-EC) were directly labeled using iron oxide nanoparticles. **(A)** MRI imaging to track the directly loaded nanoparticle cell label *(A/left)* Serial *in vivo* MR [gradient-recalled echo (GRE)] images of iron oxide nanoparticles. No hypointense signal was found in control animals injected with unlabeled cells. MR signals showed no significant difference from day 2 to day 28 (the white arrow indicates teratoma formation in the hind limb injected with hES cells). *(A/right)* Quantitative analysis of GRE signals from all animals transplanted with hES cells and hESC-ECs [signal activity is expressed as authority unit (AU)]. **(B)** Tracking of the cells by virtue of reporter gene imaging (indirect cell labeling). *(B/left)* Planar bioluminescence imaging reveals differences in signals obtained from hind limbs that received either hES or hESC-EC cells. After initial similar signal decreases in both limbs, the signals from limbs with hES increased significantly over time, coinciding with teratoma formation in these limbs. *(B/right)* Quantification of 2D bioluminescence signals from each limb (photons/sec/cm^2^/sr; note the log10 scale). **(C**, top) Immunohistochemical (IHC) analysis of initially double labeled hES cells and hESC-ECs clearly reveals iron oxide (by Prussian Blue) co-localizing with a macrophage stain (by specific antibody Mac-3); IHC counterstains were Nuclear Fast Red and Hematoxylin, respectively. Note that macrophages loaded with iron particles can be found in between muscle bundles.(**C**, bottom) Immunofluorescence staining of GFP for transplanted luciferase co-expressing hESC-ECs (left) or hESC (right). Other panels show respective counterstains for microvasculature (CD31) or macrophages (Mac-3); nuclei were stained with DAPI (blue) in merged images. All images are from four weeks after transplantation. There were no transplanted GFP^+^ hESC-ECs found nearby macrophages. In tissues that received hES cells, GFP^+^ hESC were found to form teratoma (#) but no Prussian Blue-stained nanoparticles were found in corresponding IHC regions. The dashed line separates teratoma from normal muscle fibers (*). All scale bars are 20 μm. (Figure modified with permission from Li et al., 2008).

Stem cell tracking experiments conducted with cells that were both iron oxide nanoparticle-labeled (“direct” cell label) and expressing luciferase and the fluorescent protein GFP (“indirect” reporter genes) elegantly demonstrated the differences between the two different cell labeling methodologies (Figure 6). Even though MRI signals from the iron oxide nanoparticle were detectable for four weeks, these signals were found through *ex vivo* validation by histology to stem from resident macrophages that had phagocytosed the nanoparticles, which were released from dying stem cells. In contrast, luciferase signals were recorded only from living cells and were validated *ex vivo* also by histology (Li et al., 2008). This study highlighted comprehensively that reporter gene imaging much better reflects cell viability and that great care must be taken to avoid ascribing signals from directly labeled cells to the wrong cell populations. Consequently, employing direct cell labeling necessitates independent cross-validation, which could include e.g. *in vivo* co-tracking by reporter gene imaging and *ex vivo* validation by histology or flow cytometry.

Within indirect cell tracking, there can be variations in how reporter genes reflect cell viability. Differences can arise due to the steady-state concentrations of certain reporter proteins in cells, determined by the production and degradation rate of the reporter. These turnover parameters have not been systematically studied for most reporter genes except some fluorescent proteins (Khmelinskii et al., 2016). In certain conditions, turnover can be manipulated, for example through genetic modification with oxygen degradation domains to speed up reporter degradation in normoxic conditions (Goldman et al., 2011; Misra et al., 2017). In general, both fluorescent proteins and reporters relying on contrast agent binding are likely to produce signals if present. Dying traceable cells or cell debris from them will remain detectable until the reporter proteins are cleared or destroyed. In contrast, reporters with enzyme or transporter functions need to be active to generate contrast in cells, and this requires a form of cellular energy to drive the transport. For example in the case of NIS, the Na^+^/K^+^ gradient (Dohan et al., 2003) is critical and its breakdown results in loss of NIS transporter activity, which is the basis for the high sensitivity of NIS to cell death. Consequently, if cell viability is central to the goals of a study, an activity-dependent reporter may yield more reliable data than a reporter which signal relies merely on protein presence.

### Impact of Cell Labeling Methodology on Cell Function

**FIGURE 7:**
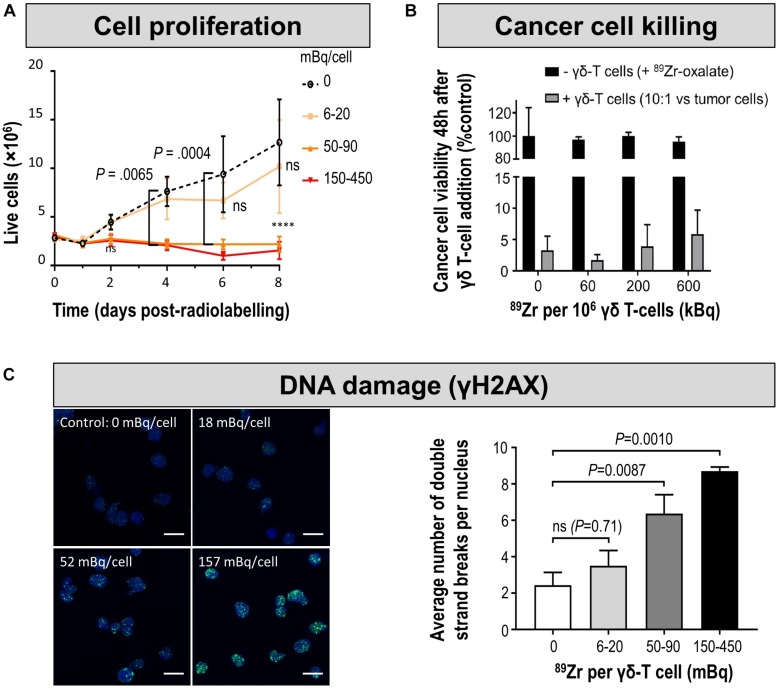
Cell characterization after direct labeling of T-cells with [^89^Zr]Zr-oxine. **(A)** *In vitro* proliferation of differently radiolabeled human γδ T-cells demonstrates that with higher amounts of cell label per cell, the capacity to proliferate diminishes. As expansion capacity is crucial for cell-based immunotherapy applications, it is paramount to perform such proliferation assays for sufficiently long times and quantify any differences even if they happen several days after cell labeling. **(B)** Tumor cell killing assay demonstrates that even γδ T-cells containing radioactivity levels incompatible with further expansion still retain at least part of their tumor killing function if supplied in sufficiently high amounts. Here, the authors reported this using a triple negative breast cancer cell line *in vitro* by quantifying tumor cell viability 48 h after immune cell addition. Notably, unchelated ^89^Zr supplied to tumor cells did not kill them and served as one of the controls. **(C)** *(left)* DNA damage analysis in radiolabeled human γδ T-cells. Representative images of γ-H2AX foci (green) and nuclei (blue); scale bars are 10 μm. *(right)* Cumulative data from the quantification of γ-H2AX foci per nuclei after radiolabelling. For statistical analysis of all data see Man et al. (2019), from where this figure is reproduced with modification and permission.

It is obvious that there should be no impact of the cell labeling methodology on the function and long-term fate of the labeled cell. Contrast agents for direct cell labeling that are compatible with highly sensitive *in vivo* detection of labeled cells are often radiotracers. While chemical/biological toxicity is mostly irrelevant due to very low tracer-level concentrations (picomolar), they have the potential to exert radio-damage to the cells depending on their cellular concentration and location, their half-life and type of radioactive decay. For example, despite their short range the Auger electrons emitted by ^111^In and to a lesser extent by ^99m^Tc have the potential to exert significant DNA damage if they come in close proximity with DNA within the cell nucleus (Sahu et al., 1995). Consequently, cell labeling with agents directly releasing them into the cytosol such as [^111^In]In-oxine could be more harmful than agents linking them to the cell surface. However, systematic comparative and quantitative studies on such radiobiological effects are not yet available in lymphocytes. Nevertheless, it can be safely assumed that great care must be taken when radiolabelling e.g. T-cells with radiometals, because irradiation is a successful method to deplete the immune system of lymphocytes indicating their distinct sensitivity to radiation (Manda et al., 2012; Piotrowski et al., 2018). Generally, the longer the half-life the larger the dose the labeled cells receive; and this is also valid for their *in vivo* environment which experiences crossfire from the labeled cells. Consequently, careful consideration of dosimetry is required as well as biological evaluation of radiation effects in radiolabeled cells. For example, [^89^Zr]Zr-oxine labeled γδ T-cells were tracked to NIS-reporter gene expressing tumors visualized by ^99m^TcO_4_^–^ in breast xenograft murine models to determine if the immunostimulatory drug alendronate would result in enhanced tumor targeting by the administered γδ T-cells (Man et al., 2019). To achieve this, the authors first titrated cellular radioactivity amounts delivered into γδ T-cells and validated its impact on γδ T-cell viability/proliferation, occurrence of DNA double strand breaks and retention of tumor cell killing function (Figure 7). This resulted in an optimized radiolabelling regimen that was then used for *in vivo* cell tracking. Using a similar approach, others determined tumor targeting and tumor retention of [^89^Zr]Zr-oxine-labeled CAR-T in a glioblastoma and prostate cancer animal model (Weist et al., 2018). The reported tolerated radioactivity levels in labeled cells were 20 mBq/cell for γδ T-cell and 70-80 mBq/cell for effector T-cells/CAR-T in these studies. Fundamentally, the tolerated radioactivity amounts limit the possible tracking time for such labeled cells.

As indirect cell tracking is based on repeat administration of short half-life radioisotopes (Figure 3C), total received doses are lower compared to direct cell labeling-afforded cell tracking over equivalent time spans. While radio damage is likely less of concern in this context, there are currently no systematic studies on radio damage of reporter-expressing lymphocytes incubated repeatedly with short half-life radiotracers available.

Another important aspect relates to the question of whether there is any impact of the typically very small administered radiotracer amounts (“tracer levels,” “microdoses”) on the corresponding target biology/physiology; in the context of this article for example whether imaging affects adoptively transferred immunotherapies. Generally accepted is the use of tracer level amounts, whereby a microdose is defined as “less than 1/100 of the dose of a test substance calculated (based on animal data) to yield a pharmacological effect of the test substance with a maximum dose of ≤100 μg or, in the case of biological agents, ≤30 nmol” (European Medicines Agency, 2004; VanBrocklin, 2008). However, there are studies available now, which should serve as a primer to investigate this matter more closely as molecules emerged that have biological effects at administered doses comparable to what is generally accepted as tracer level/microdose amounts. First, in a study aimed at radiotherapeutic evaluation of the human somatostatin receptor (hSSTr2) agonist [^90^Y]Y-DOTATOC, it was found that the agonist impaired immune function in humans (Barsegian et al., 2015). Hence, it cannot be ruled out at this point that other somatostatin-related imaging agents also have effects on the immune system, and consequently this imaging agent might not be suitable to *in vivo* track hSSTr2 reporter expressing adoptively transferred T-cells. Moreover, a recent study reported that immunoPET performed to *in vivo* track adoptively transferred T-cells in tumor-bearing mice impacted on immunotherapy outcome (Mayer et al., 2018). The authors used ^89^Zr-DFO-conjugated anti-CD7 and anti-CD2 antibody fragments (F(ab’)_2_), respectively, to quantify adoptively transferred T-cell populations in tumors. While they did not find any impact of both imaging tracers on T-cells *in vitro*, they found that the anti-CD2 radiotracer caused severe T-cell depletion and abrogated the effects of the adoptively transferred T-cell immunotherapy (the anti-CD7 radiotracer performed as expected and had no impact on adoptively transferred T-cells). The amounts of radiolabeled antibody fragment used in this study were ∼9 μmol/kg [1 mg/kg F(ab’)_2_], which was, for example, about five times less compared to what was used in the seminal immunoPET study that originally developed the anti-CD8 *cis*-diabody (∼50 μmol/kg ≈ 3 mg/kg), which is now in clinical development (see Section “*In vivo* Imaging of T-Cell Populations”). From these two examples follows clearly that great care must be taken with imaging agents in the context of immunology even when used at amounts generally accepted to be in the range of what normally constitutes tracer levels/microdoses, because there can be effects on immune cells and their functions *in vivo*, depending on the chosen imaging target. Furthermore, this is strong evidence that *in vitro* experimentation might not be indicative of such effects. Moreover, this is also an argument for the need of careful comprehensive *in vivo* validation experiments in relevant animal models during the development of imaging agents, irrespective of whether envisaged to aid immunotherapy development or intended for future immunotherapy monitoring in the clinics.

### Immunogenicity and Contrast Are Linked in Reporter Gene Applications

**FIGURE 8:**
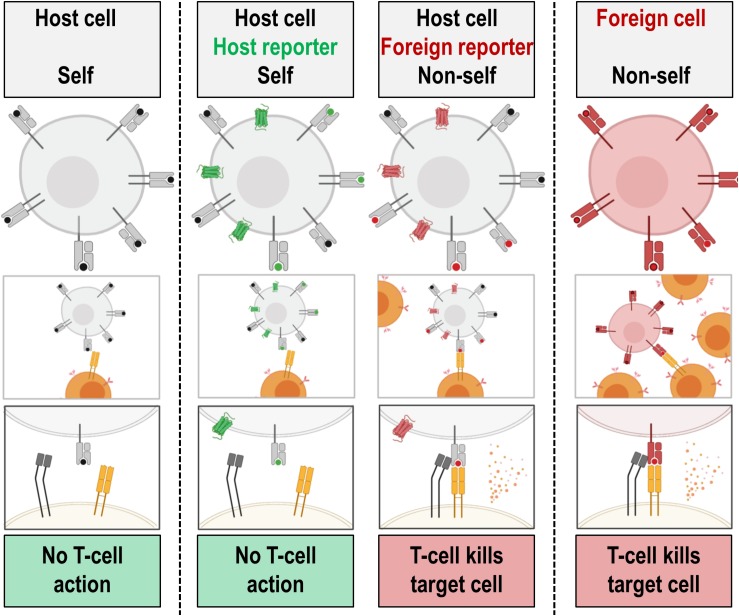
Simplified cartoon illustrating one way of cytotoxic T-cells to recognize foreign reporter antigens. A variety of immune recognition mechanisms exist in mammals as part of their innate and adaptive immune system. Here, as a simplified example, recognition of antigen-presenting MHC class I molecules on target cells by a cytotoxic CD8^+^ T-cells is visualized. The TCR (orange) of the cytotoxic CD8^+^ T-cells recognizes foreign antigen presented on host MHC class I molecules (light gray with red foreign antigen) but not host antigen on host MHC class I molecules (green: antigen from host reporter; black: any other host antigen). Foreign MHC class I molecules are also recognized by CD8^+^ T-cells. The T-cell co-receptor CD8 (dark gray) binds to MHC class I molecules upon TCR binding and the overall process activates CD8^+^ T-cells. CD8^+^ T-cell action results in granzyme and perforin release, and consequent killing of the corresponding target cell. Several mechanisms ensure that host antigens are not recognized; they include deletion of self-recognizing T-cells and tolerance conferred by regulatory T-cells. This simplified scheme demonstrates the importance to employ host reporters in experiments involving species with intact adaptive immunity.

For *in vivo* tracking of cell-based immunotherapies using reporter genes, immunogenicity of the reporter represents another very important aspect. It is linked to the achievable contrast at different body locations, which we explain in the following. For best contrast, a foreign reporter would appear ideal as it is expressed nowhere in the host organism guaranteeing good contrast. In animal disease models, such reporters are, for example, fluorescent proteins, luciferases (Mezzanotte et al., 2017) or the PET reporter herpes simplex virus 1 thymidine kinase (HSV1-*tk*) (Gambhir et al., 2000; Yaghoubi and Gambhir, 2006; Likar et al., 2009). All of them provide excellent contrast *in vivo* with varying sensitivities and spatial resolutions depending on the imaging modality used to probe them (cf. Figure 1). However, all of them are proteins foreign to mammals and consequently any cell expressing them in mammals can be detected and cleared by an intact host immune system (Figure 8). While this might represent only a minor issue if heavily immunocompromised animals are used, for example in human tumor xenograft models, it cannot be ignored in syngeneic models or the human clinical setting. While the foreign reporter (HSV1-*tk*) was used in the first proof-of-principle clinical reporter gene imaging study (Keu et al., 2017), this was performed in the setting of late-stage glioblastoma in heavily pre-treated patients, all of whom died within a year of the study start. In fact, immunogenicity of HSV1-*tk* has been well documented (Berger et al., 2006) and consequently HSV1-*tk* has been ruled out for reporter gene-afforded routine cell tracking of adoptive cell-based immunotherapies in humans; it should also not be considered for research in preclinical syngeneic models.

Immunogenicity issues can best be overcome by using host reporter proteins (Table 2) that are normally endogenously expressed in the organism of interest. Importantly, these host reporters should be endogenously expressed in only a very limited number of host tissues, only in tissues where signals do not interfere with the experimental goals, and ideally at low levels to ensure favorable contrast in adjacent organs (cf. different background patterns in Figure 5).

Mammalian NIS has been found to be useful as a radionuclide reporter if used together with non-iodine radiotracers, which results in better signal-to-background (Diocou et al., 2017). Generally in mammals, NIS is endogenously expressed at high levels in the thyroid gland and at lower levels in few extrathyroidal tissues (salivary glands, mammary glands, stomach and small intestine, testes) (Portulano et al., 2014). This means that for cell tracking applications in other organs the host reporter gene NIS provides excellent signal-to-background ratios when exogenously expressed in cells of interest. NIS has been used to track many different cell types preclinically (Sieger et al., 2003; Groot-Wassink et al., 2004; Che et al., 2005; Dingli et al., 2006; Merron et al., 2007; Terrovitis et al., 2008; Carlson et al., 2009; Higuchi et al., 2009; Fruhwirth et al., 2014; Diocou et al., 2017) including stem cell and CAR-T-cell therapies (Emami-Shahri et al., 2018; Kurtys et al., 2018; Ashmore-Harris et al., 2019). It has not yet been used in the clinic but due to its favorable properties and the readily available corresponding radiotracers for both PET and SPECT for its detection, it is a promising candidate for use in future clinical trials.

The human somatostatin receptor subtype 2 (hSSTr2) is another reporter with some potential for cell tracking using clinically approved PET tracers based on somatostatin analogs [e.g. [^68^Ga]Ga-DOTATATE (antagonist) or [^68^Ga]Ga-DOTATOC (agonist)] and it has been used preclinically for CAR-T tracking (Zhang et al., 2011; Vedvyas et al., 2016). A significant pitfall of hSSTr2 use as a reporter for immunotherapies is that it is expressed endogenously on various immune cell types including T-cells, B-cells and macrophages (Elliott et al., 1999). This negatively affects imaging specificity in immunocompetent models and likely humans. It is also expressed in the cerebrum, kidneys and also the gastrointestinal tract (Yamada et al., 1992). Moreover, it was found that the hSSTr2 agonist [^90^Y]Y-DOTATOC impaired immune function in humans (Barsegian et al., 2015). Whilst radioactive contrast agent concentrations are very low, it cannot be ruled out without further studies that somatostatin analogs and their derivatives might also impair some immune system functions. Another important caveat of hSSTr2 use as a reporter is that it internalizes upon substrate binding (Oomen et al., 2001; Cescato et al., 2006) and this is likely to affect the detection sensitivity of hSSTr2-expressing cells through reduction of its steady-state concentration on the plasma membrane.

A very promising host reporter gene with very limited endogenous expression is PSMA (Castanares et al., 2014). It has been developed alongside PET radiotracers that were originally intended for molecular imaging of PSMA-expressing prostate cancer. PSMA is a type II plasma membrane protein that can be internalized upon ligand binding. It has a short cytoplasmic N-terminal tail, which is responsible for its internalization (Rajasekaran et al., 2003). N-terminally modified PSMA variants, PSMA^W2G^ and tPSMA^N9del^, were recently designed to prevent receptor internalization and to increase PSMA surface expression with the authors hypothesizing that would increase PET radiotracer binding and overall imaging sensitivity (Minn et al., 2019). Moreover, the tPSMA^N9del^ variant lacks putative intracellular signaling motifs rendering it less likely to affect normal T-cell function. tPSMA^N9del^ was used as a reporter to track CAR-T cells in a preclinical model of acute lymphoblastic leukemia by PET imaging with the radiotracer [^18^F]DCFPyL (Minn et al., 2019). [^18^F]DCFPyL is a radiotracer for PSMA, in fact a high-affinity PSMA ligand that can be produced in good quantities and with high specific activity (Ravert et al., 2016), and it has already been used in humans and is currently also in a phase II clinical trial for the detection of metastatic prostate cancer *via* PSMA (NCT03173924), another application that requires the detection of small amounts of cells.

Despite some notable advances in recent years, there is still significant room for improvement to optimize host reporter/tracer pairs, for example to improve signal-to-background, tailor them better to application in specific immune cells, and enhance the steady-state concentrations in traceable cells and thereby cell tracking sensitivity.

## Conclusion and Outlook

The development of both molecular and cell-based immunotherapies can be greatly assisted by *in vivo* imaging, which provided valuable insight into spatiotemporal dynamics of immune responses and the complex interactions of the tumor microenvironment. *In vivo* imaging has earned itself a place among the indispensable tools for immunotherapy development at preclinical stages, and many available molecular imaging technologies can be used for understanding the mechanisms governing immunotherapy function and to improve immunotherapy efficacy and safety. Newly identified relevant targets will require some degree of molecular imaging development to generate the relevant contrast agent, but multiple robust methodologies for turning target-specific biomolecules such as antibodies, antibody fragments/derivatives or peptides into contrast agents are already available. Various molecular imaging techniques aiding immunotherapy are currently at the brink of clinical application, mostly still in explorative studies, some in clinical trials, and they focus on early response monitoring with response prediction representing a major goal. Individual response monitoring at the patient level is particularly important as responses can be heterogeneous between lesions within the same individual and also between patients, rendering this a potential routine clinical application of molecular imaging in the future. A currently somewhat underexplored area is immunotherapy presence and action at secondary lesions. Preclinically, traceable cancer models would be very useful tools in this context, enabling *in vivo* quantification of therapy arrival and perhaps therapy action at the intended target sites. Clinically, molecular imaging will help inform on lesion heterogeneity as well as potential response heterogeneity in patients.

Cell-based immunotherapies represent an area in need of further development to unleash their full potential and render them more efficacious, safer to use, and more widely applicable. Therefore, it remains highly beneficial to better understand their *in vivo* distribution, behavior and fate, and to use such non-invasively acquired information to elucidate and tailor their mechanisms of action. Cell-based immunotherapies can be classified into two groups that (a) do not need genetic engineering for efficacy, and those that (b) fundamentally require genetic engineering (e.g. CAR-T, TCR-T).

The first group, which includes immunotherapies based on e.g. TILs and γδ T-cells, the choice between direct and indirect cell labeling depends on the precise research question, practicalities and of course whether clinical translation of the tracking methodology is envisaged and for what purpose. Implementing genetic engineering to enable indirect cell labeling to these therapies adds a significant regulatory burden and it is certainly difficult to justify the additional efforts required for the sole purpose of *in vivo* cell therapy tracking. Consequently, recently developed direct cell labeling approaches involving cell tracking by PET (e.g. γδ T-cell labeling with [^89^Zr]Zr-oxine) are promising tools despite their obvious limitations caused by the cell labeling methodology itself (label efflux, label dilution, complex dosimetry, limited observation times). However, the situation is likely to improve through the development of total-body PET, which has been reported to be 40-times more sensitive than conventional PET (Cherry et al., 2018). This sensitivity advantage could either be invested into faster PET scanning or scanning with much less radioactivity. *In vivo* cell tracking studies using this new technology will reveal to what extent the sensitivity advantage of total-body PET can be used to extend the tracking time of directly labeled cells.

For cell-based immunotherapies that require genetic engineering, an immunocompatible host reporter gene can be implemented without adding to the regulatory burden. Indirect cell labeling is clearly advantageous over direct cell labeling in such cases as it enables longer-term monitoring, reflects cell proliferation/survival, and avoids complex dosimetry considerations during cell labeling. Genetic engineering technologies have been steadily advanced and include now viral as well as non-viral delivery methods as well as site-specific integration via gene editing approaches (Figure 3C). Moreover, the reporter gene can be co-delivered with other relevant components during genetic engineering of the cells as was previously demonstrated rendering CAR-T traceable by SPECT or PET (Emami-Shahri et al., 2018; Kurtys et al., 2018; Minn et al., 2019). If contrast agents can be used that match the reporter and are already clinically approved, this is obviously beneficial. Importantly, it is unlikely that a one-fits-all approach across cancers involving only one immunocompatible host reporter gene is viable. More likely, various cancers at different body locations with varying endogenous host reporter expression levels will be targeted by genetically engineered cell-based immunotherapies in which the targeting moiety as well as the host reporter must be tailored. Undoubtedly, more research into host reporter/contrast agent pairs is warranted to provide the most flexible tools to render these immunotherapies *in vivo* traceable with best contrast in a quantitative manner.

In summary, we described how *in vivo* imaging can aid the development of molecular and cell-based anti-cancer immunotherapies and explained a variety of methodological and experimental design aspects. Notably, these concepts can also be extrapolated to immunotherapies intended to treat other conditions, for example, in the fields of regenerative medicine (Naumova et al., 2014), transplantation (Afzali et al., 2013; Safinia et al., 2016), diabetes type I (Alhadj Ali et al., 2017; Smith and Peakman, 2018), multiple sclerosis (Chataway et al., 2018), and infectious diseases (Hotchkiss and Moldawer, 2014).

## Author Contributions

GF contributed the article concept. Both authors compiled the figures, wrote the manuscript, and contributed to manuscript revision, read, and approved the submitted version.

## Conflict of Interest

The authors declare that the research was conducted in the absence of any commercial or financial relationships that could be construed as a potential conflict of interest.

## Abbreviations

ATMP: advanced therapy medicinal products
BLI: bioluminescence imaging a preclinical imaging technology
CAR: chimeric antigen receptor an artificial molecule
CAR-T: chimeric antigen receptor T-cell therapy
CD2: cluster of differentiation 2 a cell adhesion molecule found on the surface of T-cells and natural killer cells also known as T-cell surface antigen T11/Leu-5, LFA-2 LFA-3 receptor erythrocyte receptor and rosette receptor
CD3: cluster of differentiation 3 a T-cell co-receptor
CD4: cluster of differentiation 4 a glycoprotein found on the surface of immune cells such as T helper cells, monocytes macrophages and dendritic cells
CD7: cluster of differentiation 7 found on thymocytes and mature T-cells
CD8: cluster of differentiation 8 a glycoprotein that serves as a co-receptor for the T-cell receptor predominantly expressed on the surface of cytotoxic T-cells
CD19: cluster of differentiation 19 also known as B-Lymphocyte Surface Antigen B4T-Cell Surface Antigen Leu-12 and CVID3 is a transmembrane protein
CD25: cluster of differentiation 25 also known as IL-2 receptor alpha chaina part of the high-affinity IL receptor
CT: computed tomography a whole-body imaging technology employing X–rays
CTLA-4: cytotoxic T-lymphocyte -associated protein 4 also known as cluster of differentiation 152 a molecule that is part of an immune checkpoint axis
DC: dendritic cell a type of immune cell
DFO: desferrioxamine a chelator for metal ions
FDA: U.S. Food and Drug Administration [^18^F]FHBG 9-(4-[^18^F]-Fluoro-3-[hydroxymethyl]butyl)guaninea radiotracer for the reporter gene HSV1-*tk*
FMT: fluorescence mediated tomography a fluorescence-based whole-body imaging technology
HMPAO: hexamethylene-propyleneamine oxime an agent to label cells directly with ^99m^Tc
HSV1-*tk*: Herpes Simplex Virus 1 thymidine kinase an enzyme that can be exploited as a reporter gene
ICI: immune checkpoint inhibitor
IL: IL, immune mediators specified by a number to identify the individual molecule (e.g. IL-2 and IL-12) IrAE immune related adverse event
LAG-3: lymphocyte-activation gene 3 an immune checkpoint molecule
MRI: magnetic resonance imaging an imaging technology
MSOT: multispectral optoacoustic tomography an advanced form of PAT imaging
NET: norepinephrine transporter a mammalian protein that can be used as a host reporter gene
NIS: sodium iodide symporter a mammalian protein that can be used as a host reporter gene
NK: natural killer cell a type of immune cell
OCT: optical coherence tomography an optical imaging technology
OPT: optical projection tomography a preclinical optical imaging technology
OVA: ovalbumin main protein found in egg white widely used as a model antigen in T-cell biology Numerous mouse cancer models have been modified to express OVA to aid in enhancing and tracking tumor-specific T-cell responses
PAT: photoacoustic tomography an imaging technology
PD-1: programed cell death protein 1 an immune checkpoint molecule
PD-L1: programed death ligand 1 also known as cluster of differentiation 274 or B7 homolog 1 (B7-H1) constitutes an immune checkpoint axis together with PD-1
PET: positron emission tomography a whole-body imaging technology detecting γ-photons produced by the annihilation of positrons stemming from the decay of certain radioisotopes
PSMA: prostate-specific membrane antigen a glutamate carboxypeptidase 2 also known as folate hydrolase 1
qPCR: quantitative polymerase chain reaction an analysis technique to determine specific DNA amounts in biological samples
RSOM: raster-scanning optoacoustic mesoscopy a preclinical imaging technology exploiting the photoacoustic effect
SPECT: single photon emission computed tomography a whole-body imaging technology detecting radioisotope location in 3D
TCR: T-cell receptor a multi-protein complex responsible for many T-cell activation mechanisms
TCR-T: T-cell receptor-modified T-cell therapy
TIL: tumor infiltrating lymphocyte
US: ultrasound imaging a cheap standard imaging technology.
